# Supplementary material for: Are (Co)Polymers of 1,1,3,3,3-Pentafluoropropene Possible?
Source: Molecules. 2023 Jun 7;28(12):4618. doi: 10.3390/molecules28124618 (PMC10304894; doi:10.3390/molecules28124618)
Supplement: Supplementary file 1 [file molecules-28-04618-s001.zip › molecules-2398580-supplementary.pdf]

SUPPORTING INFORMATION

Are (co)polymers of 1,1,3,3,3-pentafluoropropene possible?

*Frédéric Boschet, Georgi Kostov, Hristina Raynova, Bruno Ameduri**

Institut Charles Gerhardt, CNRS, ENSCM, Montpellier, France

bruno.ameduri@enscm.fr

Figure S1: expansion of the 0.8-3.6 ppm zone in the ^{1}H NMR spectrum of poly(PFP-*co*-VDF) recorded in acetone-d_6. (Run No 1 in Table 1). 3
Figure S2: expansion of the -59 to -76 ppm zone in the ^{19}F NMR spectrum of poly(PFP-*co*-MAF-TBE) recorded in acetone-d_6. (Run No 5 in Table 1). 4
Figure S3: ^{1}H NMR spectrum of poly(PFP-*co*-MAF-TBE) recorded in acetone-d_6. (Run No 5 in Table 1). 5
Figure S4: ^{19}F NMR spectrum of poly(PFP-*co*-HFP) recorded in acetone-d6 (Run No 2, Table 2)........ 6
Figure S5: ^{19}F NMR spectrum of poly(PFP-*co*-PMVE) cooligomer recorded in acetone-d_6 (Run No 7, Table 2)........ 7
Figure S6: ^{1}H NMR spectrum of poly(PFP-*co*-PMVE) cooligomer recorded in acetone-d_6. (Run No 7 in Table 2). 8
Figure S7: ^{19}F NMR spectrum of poly(PFP-*co*-TFP) cooligomer recorded in acetone-d6 (Run No 9, Table 2)........ 9
Figure S8: ^{1}H NMR spectrum of poly(PFP-*co*-TFP) cooligomer recorded in acetone-d_6. (Run No 9 in Table 2). 10
Figure S9: ^{19}F NMR spectrum of poly(PFP-*co*-CTFE) copolymer recorded in acetone-d6 (Run No 1, Table 4)........ 11
Figure S10: expansion of the zone between 1.2 to 3.8 ppm in the ^{1}H NMR spectrum of poly(PFP-*co*-CTFE) copolymer recorded in acetone-d_6. (Run No 1 in Table 4). 12
Figure S11: expansion of the zone between -65 to -120 ppm in the ^{19}F NMR spectrum of poly(VDF-*co*-MAFTBE) copolymer recorded in acetone-d_6. (Run No 3 in Table 3)........ 13

Figure S12: ^{1}H NMR spectrum of poly(VDF-*co*-MAF-TBE) copolymer recorded in acetone-d_6. (Run No 3 in Table 3)........ 14
Figure S 13: ^{19}F NMR spectrum of poly(VDF-*co*-HFP) copolymer recorded in acetone-d_6. (Run No 3 in Table 2). 15
Figure S14: ^{1}H NMR spectrum of poly(VDF-*co*-HFP) copolymer recorded in acetone-d_6. (Run No 3 in Table 2). 16
Figure S15: Expansion of the 0.8-3.4 ppm zone in the ^{1}H NMR spectrum of poly(PFP-*ter*-VDF-*ter*-MAF-TBE) terpolymers (recorded in acetone-d_6) (Run 6-10 in Table 3) 17
Figure S16: expansion of the 0.9-3.9 ppm zone of the ^{1}H NMR spectrum of poly(PFP-*ter*-VDF-*ter*-HFP) terpolymer recorded in acetone-d_6 (Run No 5, Table2)........ 18
Figure S17:^{1}H NMR spectrum of poly(PFP-*ter*-VDF-*ter*-PMVE) terpolymer (recorded in acetone d_6) (Run 8 in Table 2). 19
Figure S18: ^{19}F NMR spectrum of poly(PFP-*ter*-VDF-*ter*-TFP) terpolymer (recorded in acetone d6) (Run 10 in Table 2)........ 20
Figure S19:^{1}H NMR spectrum of poly(PFP-*ter*-VDF-*ter*-TFP) terpolymer (recorded in acetone d6) (Run 10 in Table 2)........ 21
Figure S20: ^{19}F NMR spectrum of poly(PFP-*ter*-CTFE-*ter*-mTMI) terpolymer (recorded in acetone d6) (Run 3 in Table 4)........ 22
Figure S21: expansion of the 0.6 to 4.0 zone in the ^{1}H NMR spectrum of poly(PFP-*ter*-CTFE-*ter*-mTMI) terpolymer (recorded in acetone d_6) (Run 3 in Table 4) 23
Figure S22: ^{19}F NMR spectrum of poly(PFP-*ter*-CTFE-*ter*-VDF) terpolymer (recorded in acetone d6) (Run 2 in Table 4)........ 24
Figure S23:^{1}H NMR spectrum of poly(PFP-*ter*-CTFE-*ter*-VDF) terpolymer, recorded in recorded in acetone d_6) (Run 2 in Table 4) 25
Figure S24: ^{1}H NMR spectrum of poly(PFP-*ter*-CTFE-*ter*-VCA) terpolymer (recorded in acetone d_6) (Run 4 in Table 4) 26
Figure S25: ^{19}F NMR spectrum of poly(PFP-*ter*-CTFE-*ter*-EVE) terpolymer (recorded in acetone d6) (Run 5 in Table 4)........ 27
Figure S26:^{1}H NMR spectrum of poly(PFP-*ter*-CTFE-*ter*-EVE) terpolymer (recorded in acetone d_6) (Run 5 in Table4) 28
Figure S27: GPC chromatogram (Solvent THF, PMMA standards) of poly (PFP-*ter*-VDF-*ter*-MAF-TBE) terpolymers. The run numbers correspond to experiment numbers in Table 3.... 29
Figure S28: GPC chromatograms of co- and ter-polymers based on PFP, VDF and HFP...... 30
Figure S29: TGA thermograms under nitrogen of PFP/VDF, PFP/MAF-TBE, VDF/MAF-TBE copolymers. The number of the experiments of the caption corresponds to the number of experiments in Tables 1 and 3........ 31
Figure S30: TGA thermograms under air of poly(PFP-*co*-VDF), poly(PFP-*co*-MAF-TBE), and poly(VDF-*co*-MAF-TBE) copolymers. The number of the experiments of the caption corresponds to the number of experiments in Tables 2-3. 32
Figure S31: TGA thermograms under nitrogen of poly(PFP-*ter*-VDF-*ter*-MAF-TBE) terpolymers. The number of the experiments of the caption corresponds to the number of experiments in Table 1........ 33
Figure S32: TGA thermograms under air of poly(PFP-*ter*-VDF-*ter*-MAF-TBE) terpolymers. The number of the experiments of the caption corresponds to the number of experiments in Table 1........ 34
Figure S33: TGA thermograms under air of poly(PFP-*ter*-VDF-*ter*-HFP), co- and ter-polymers (Table 2). 35

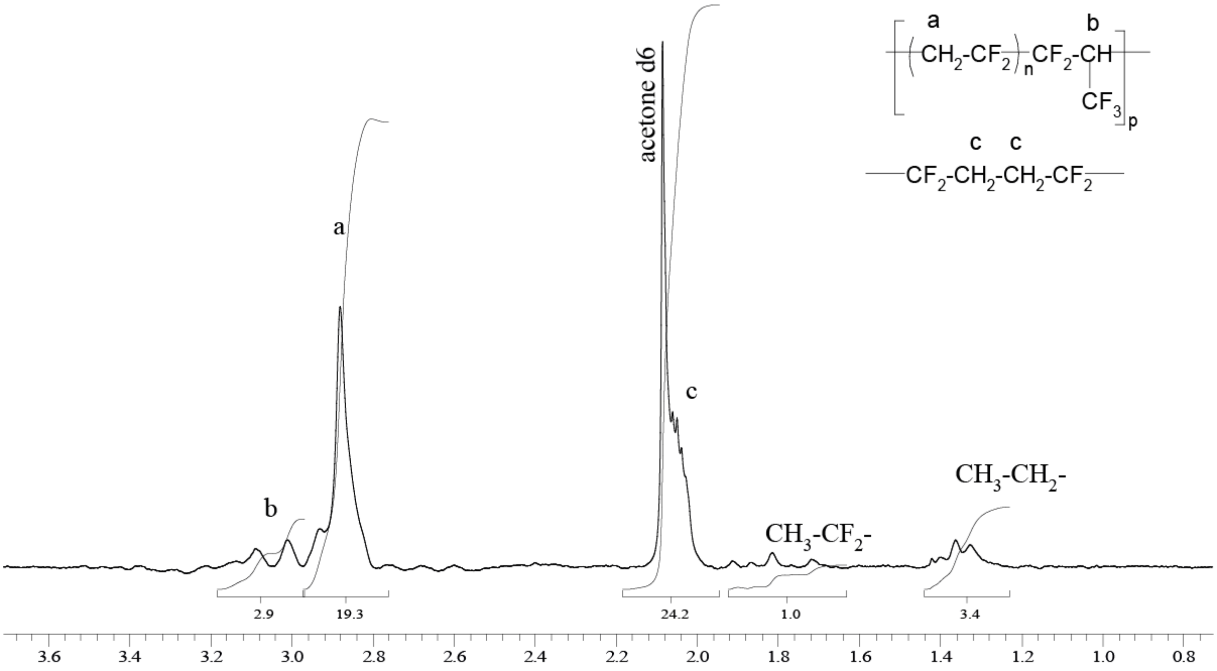

Figure S1: expansion of the 0.8-3.6 ppm zone in the ^{1}H NMR spectrum of poly(PFP-*co*-VDF) recorded in acetone-d_6. (Run No 1 in Table 1).

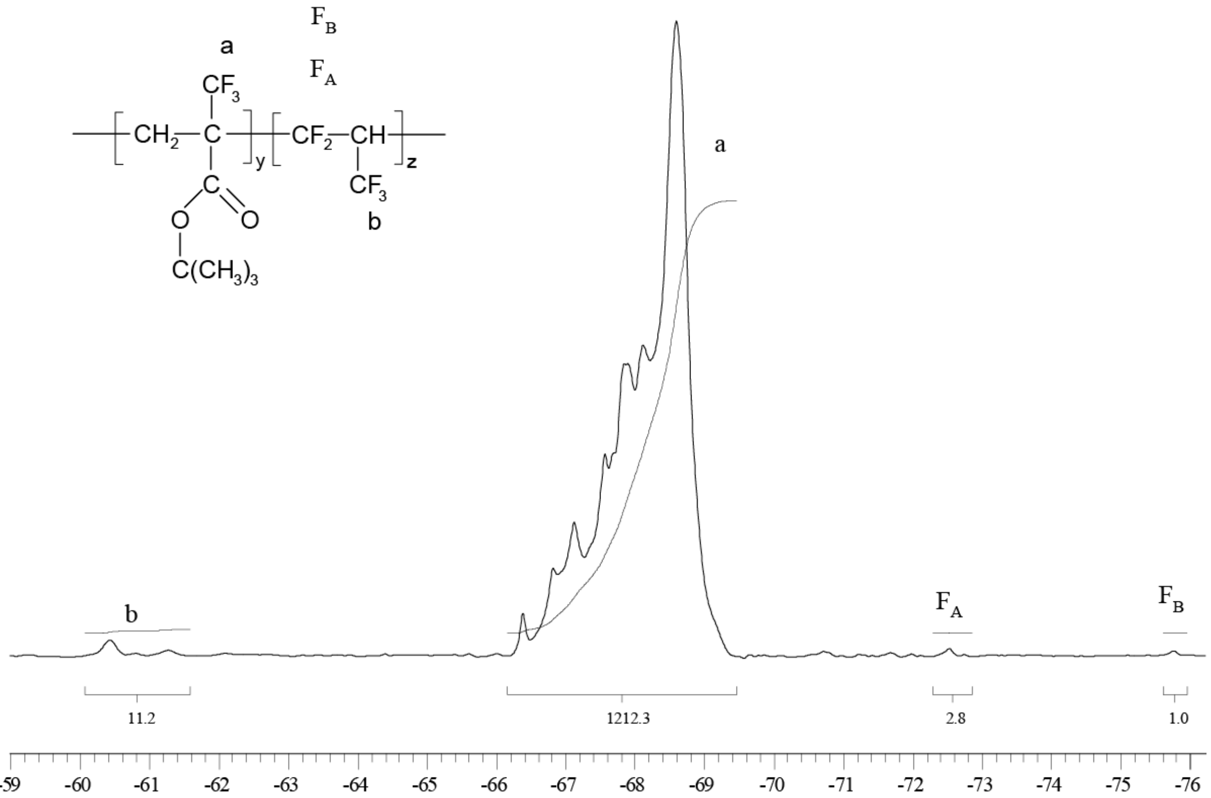

Figure S2: expansion of the -59 to -76 ppm zone in the ^{19}F NMR spectrum of poly(PFP-*co*-MAF-TBE) recorded in acetone-d_6. (Run No 5 in Table 1).

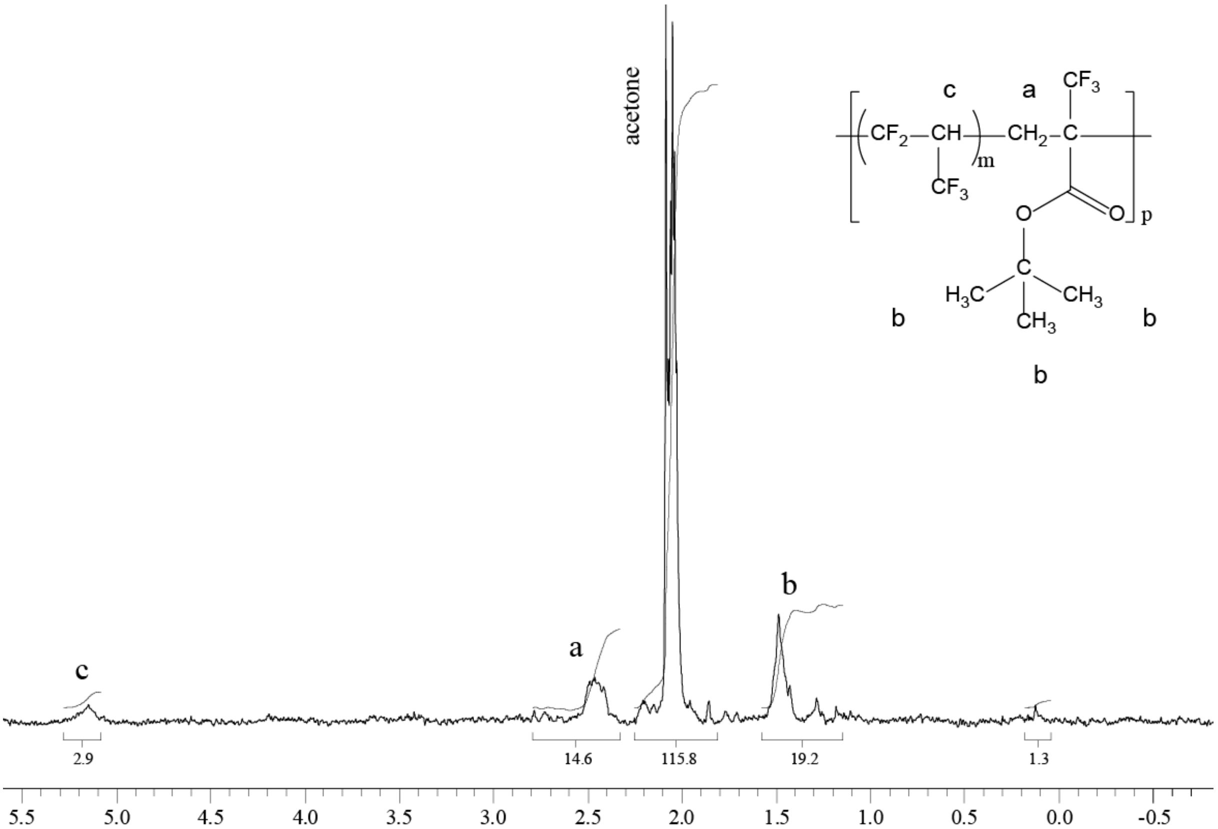

Figure S3: ^{1}H NMR spectrum of poly(PFP-*co*-MAF-TBE) recorded in acetone-d_6. (Run No 5 in Table 1).

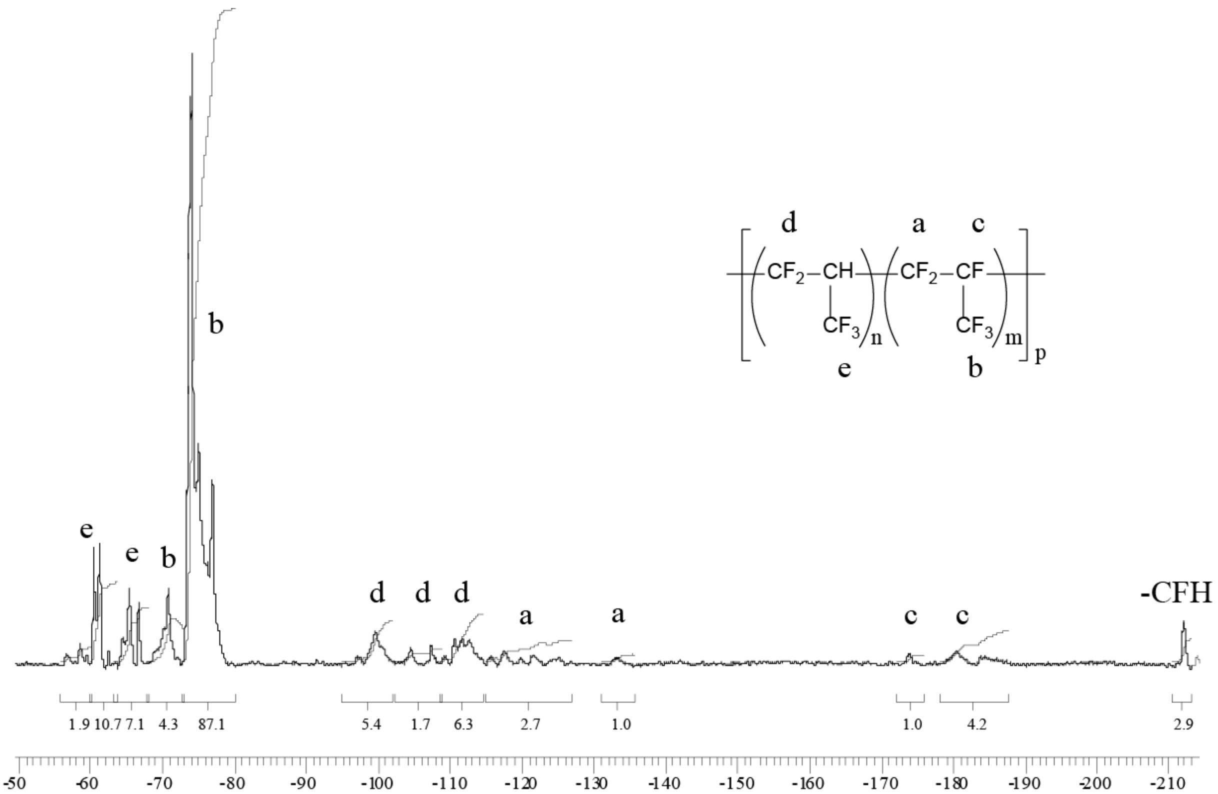

Figure S4: ^{19}F NMR spectrum of poly(PFP-*co*-HFP) recorded in acetone-d6 (Run No 2, Table 2)

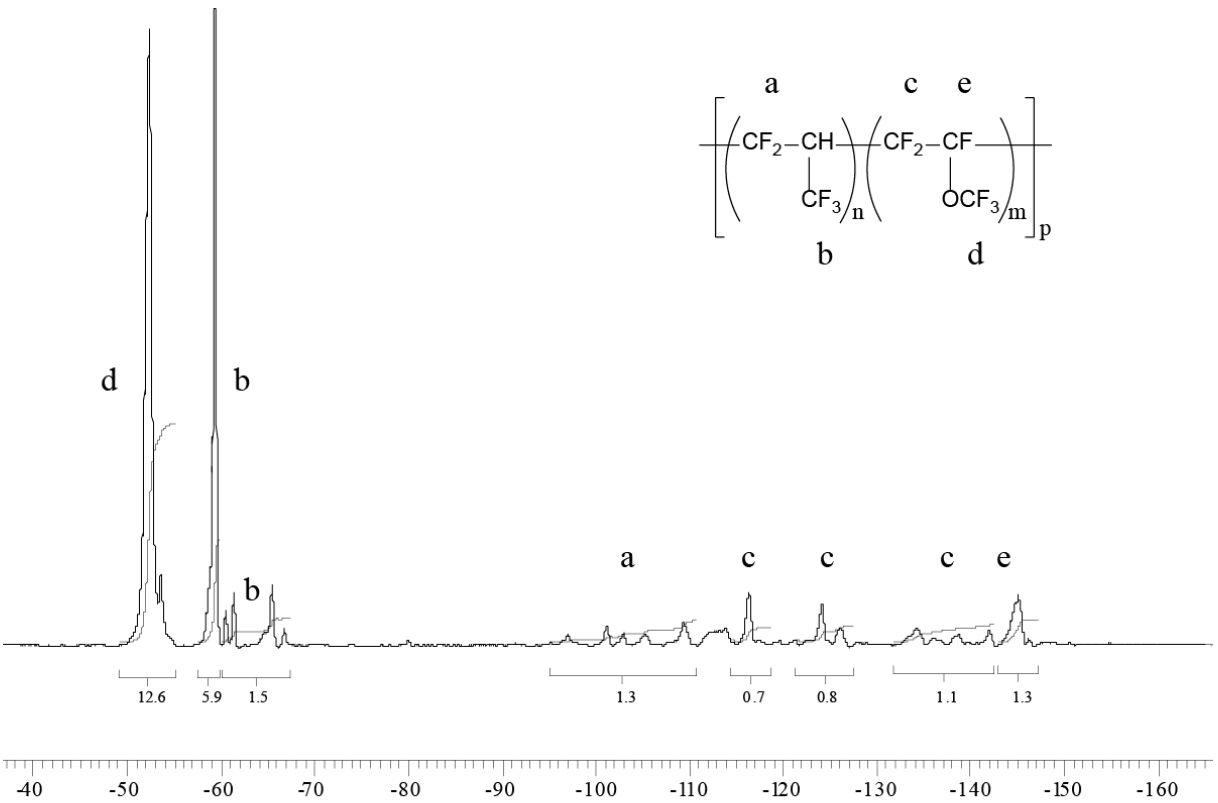

Figure S5: ^{19}F NMR spectrum of poly(PFP-*co*-PMVE) cooligomer recorded in acetone-d_6 (Run No 7, Table 2)

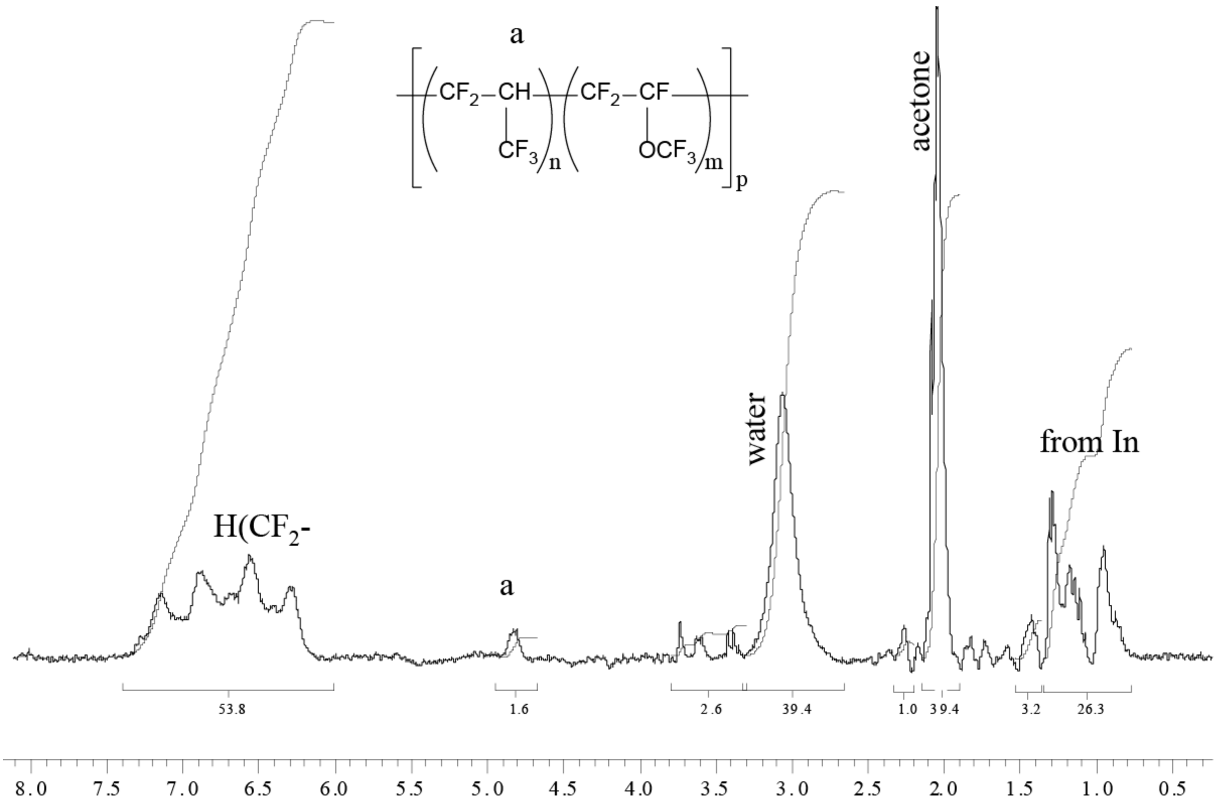

Figure S6: ^{1}H NMR spectrum of poly(PFP-*co*-PMVE) cooligomer recorded in acetone-d_6. (Run No 7 in Table 2).

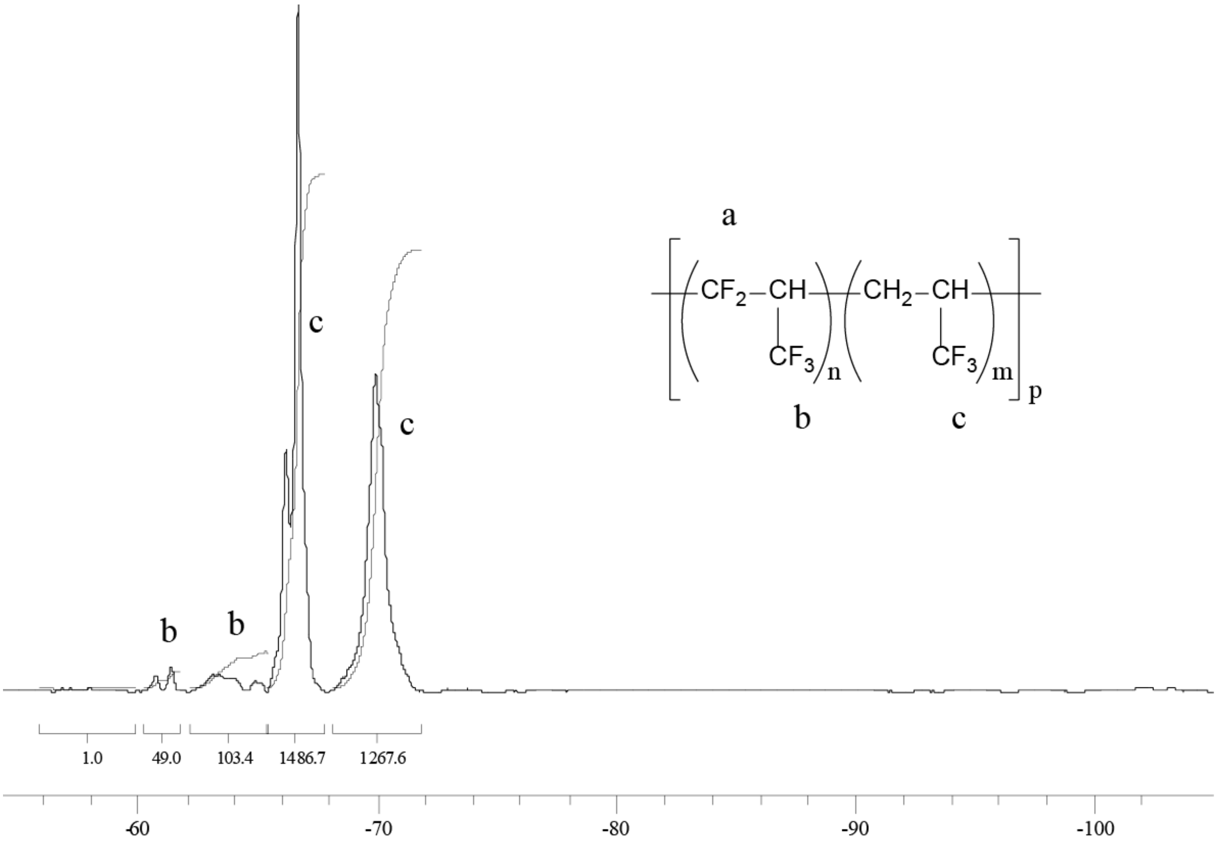

Figure S7: ^{19}F NMR spectrum of poly(PFP-*co*-TFP) cooligomer recorded in acetone-d6 (Run No 9, Table 2)

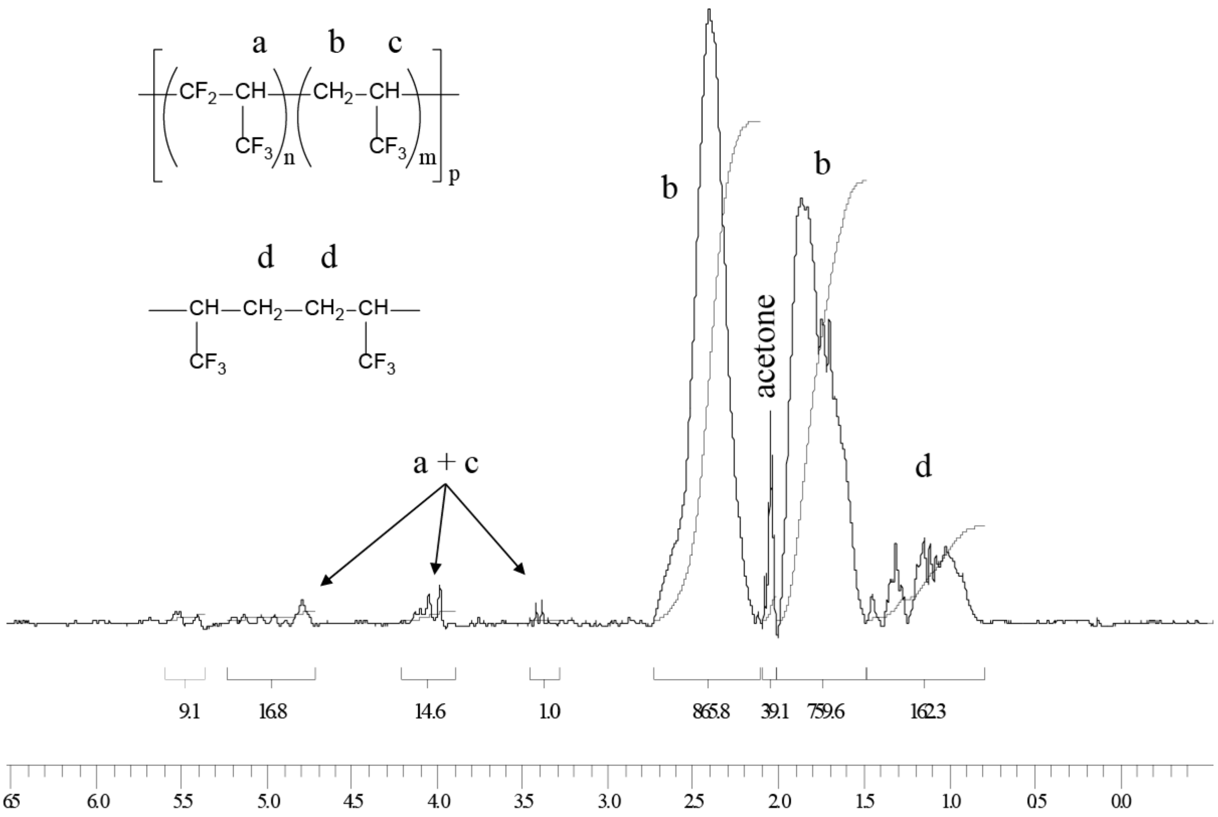

Figure S8: ^{1}H NMR spectrum of poly(PFP-*co*-TFP) cooligomer recorded in acetone-d_6. (Run No 9 in Table 2).

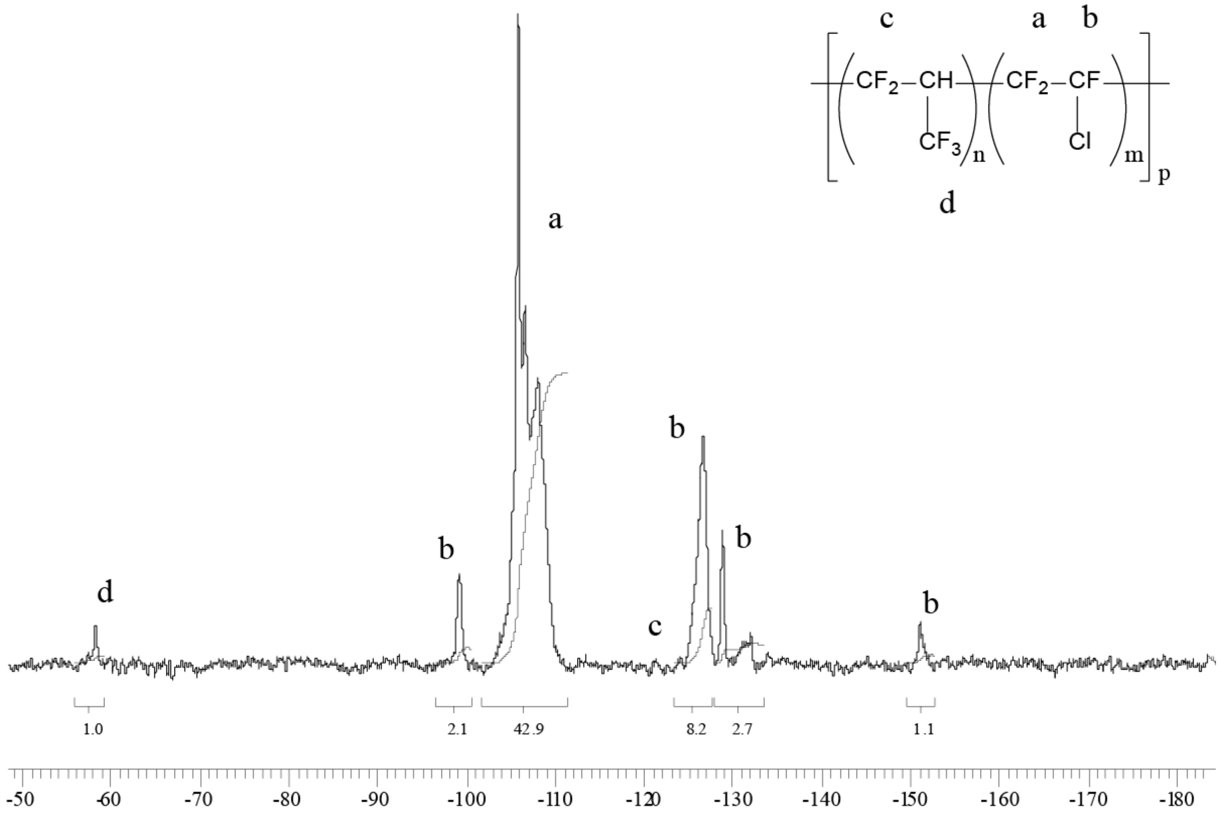

Figure S9: ^{19}F NMR spectrum of poly(PFP-*co*-CTFE) copolymer recorded in acetone-d6 (Run No 1, Table 4)

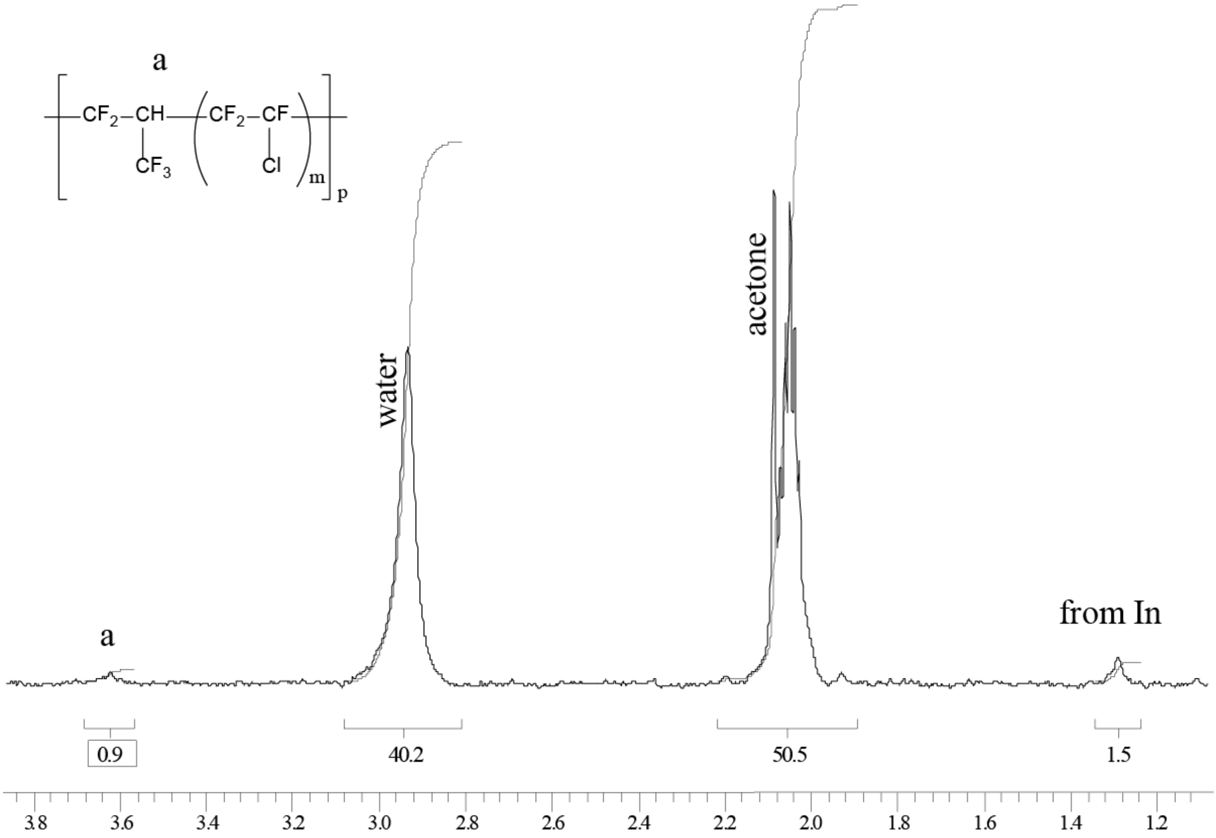

Figure S10: expansion of the zone between 1.2 to 3.8 ppm in the ^{1}H NMR spectrum of poly(PFP-*co*-CTFE) copolymer recorded in acetone-d_6. (Run No 1 in Table 4).

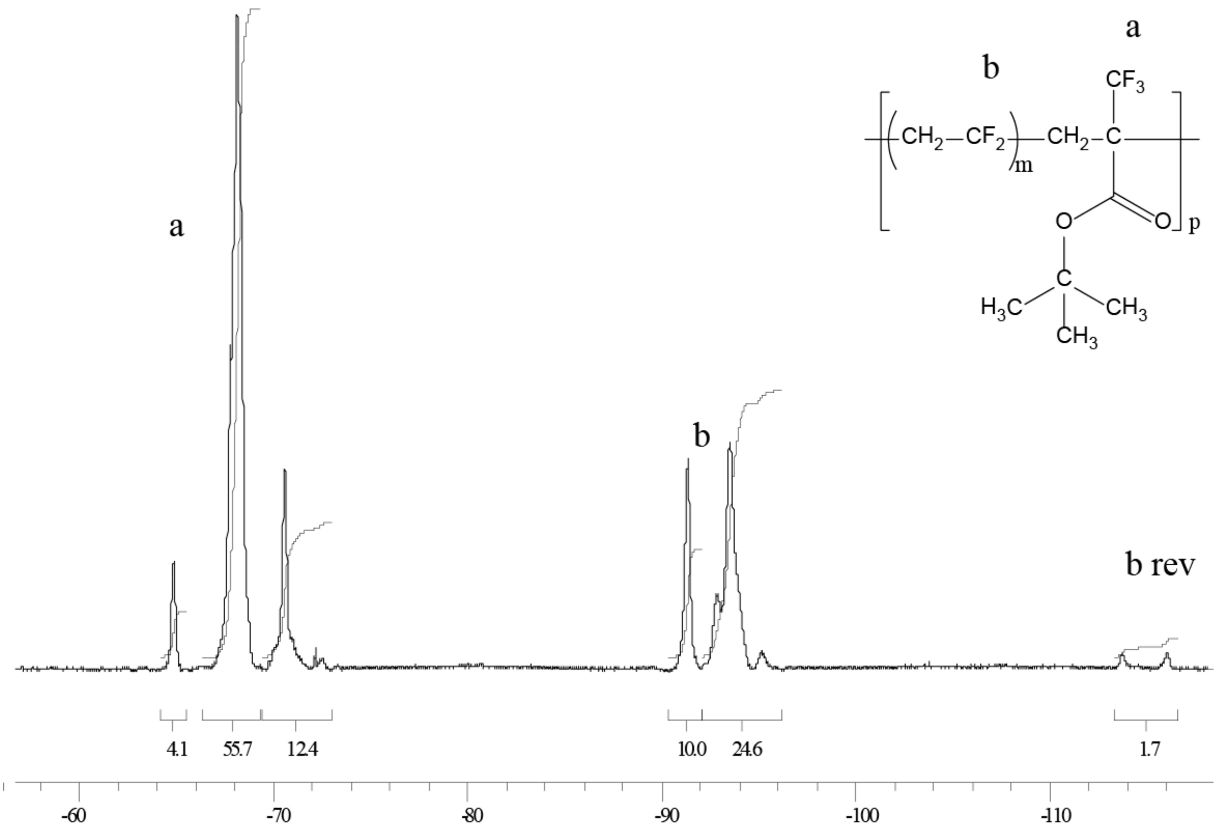

Figure S11: expansion of the zone between -65 to -120 ppm in the ^{19}F NMR spectrum of poly(VDF-*co*-MAFTBE) copolymer recorded in acetone-d_6. (Run No 3 in Table 3).

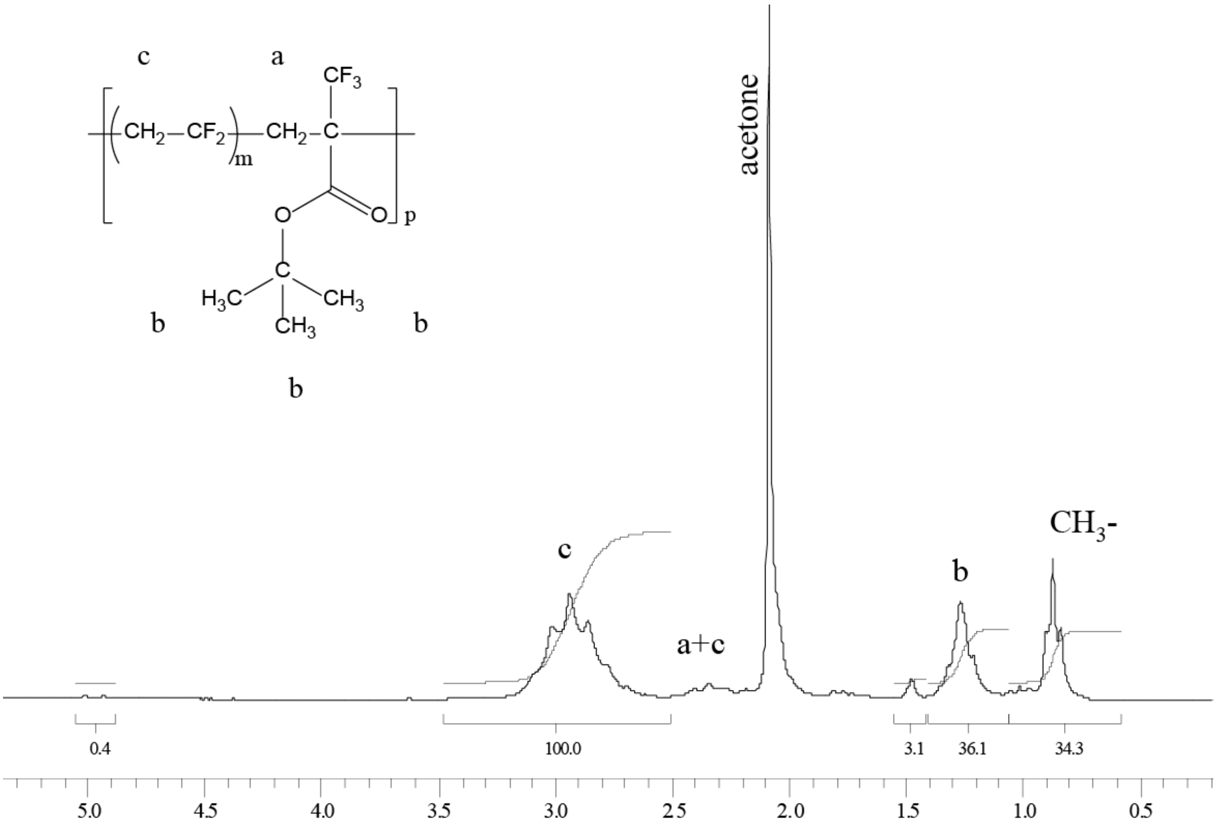

Figure S12: ^{1}H NMR spectrum of poly(VDF-*co*-MAF-TBE) copolymer recorded in acetone-d_6. (Run No 3 in Table 3).

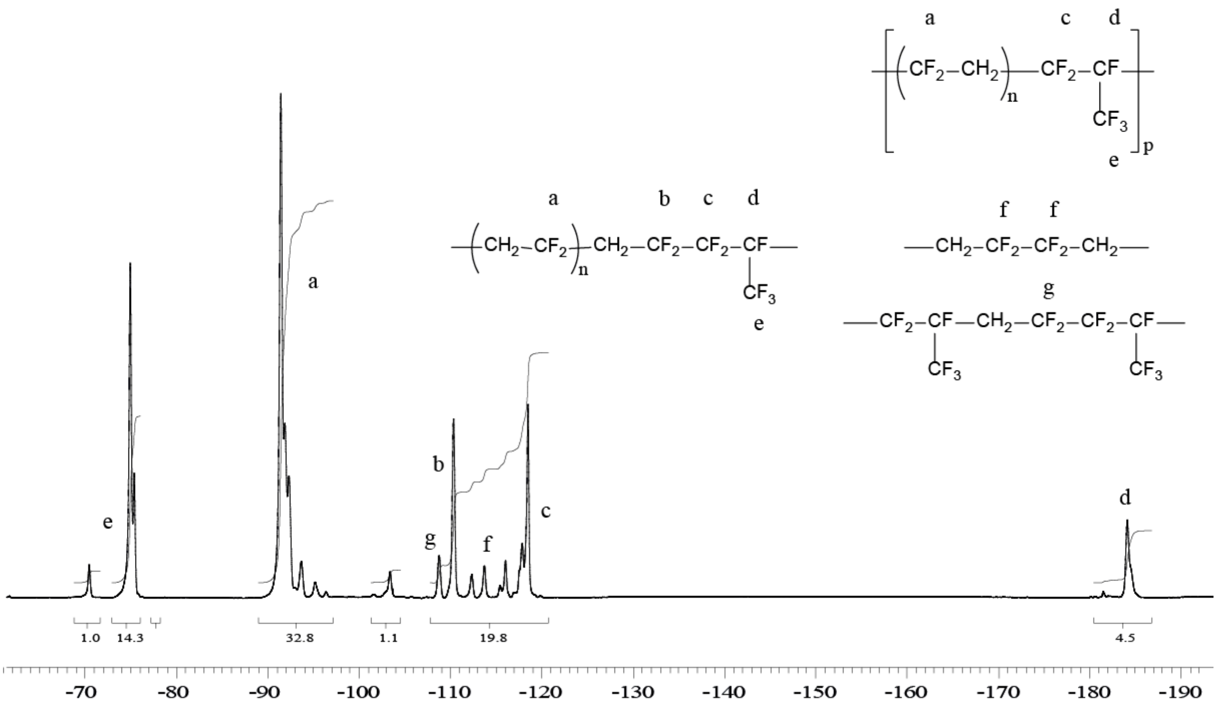

Figure S13: ^{19}F NMR spectrum of poly(VDF-*co*-HFP) copolymer recorded in acetone-d_6. (Run No 3 in Table 2).

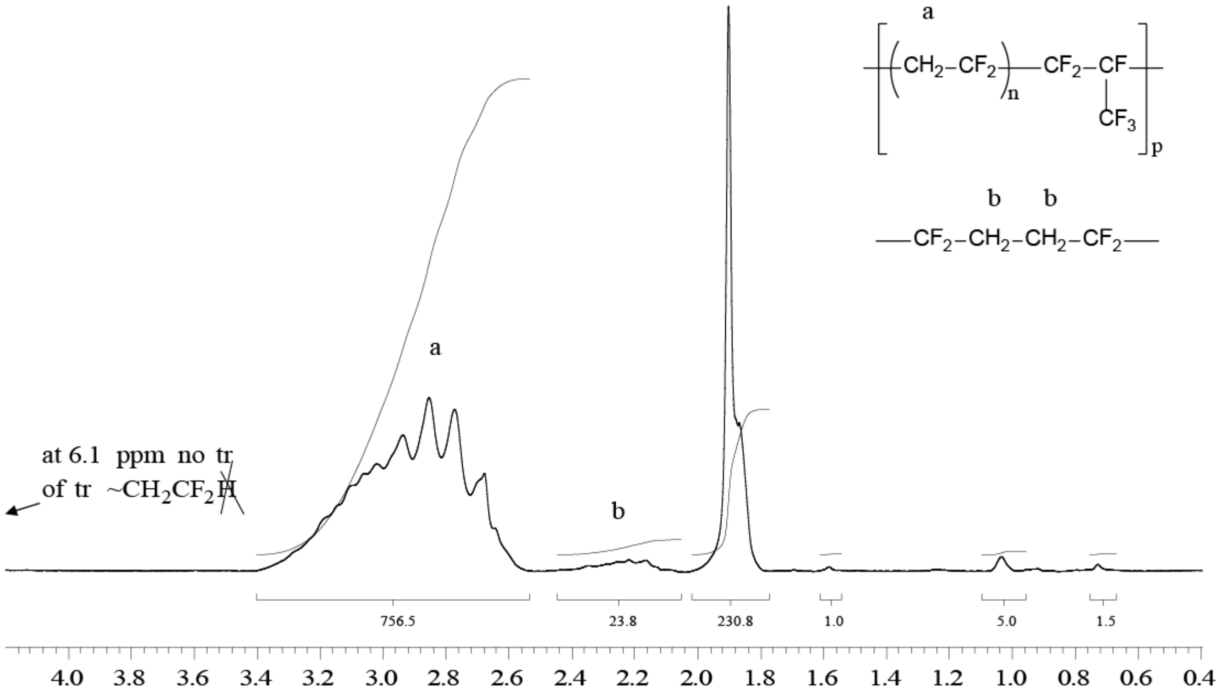

Figure S14: ^{1}H NMR spectrum of poly(VDF-*co*-HFP) copolymer recorded in acetone-d_6. (Run No 3 in Table 2).

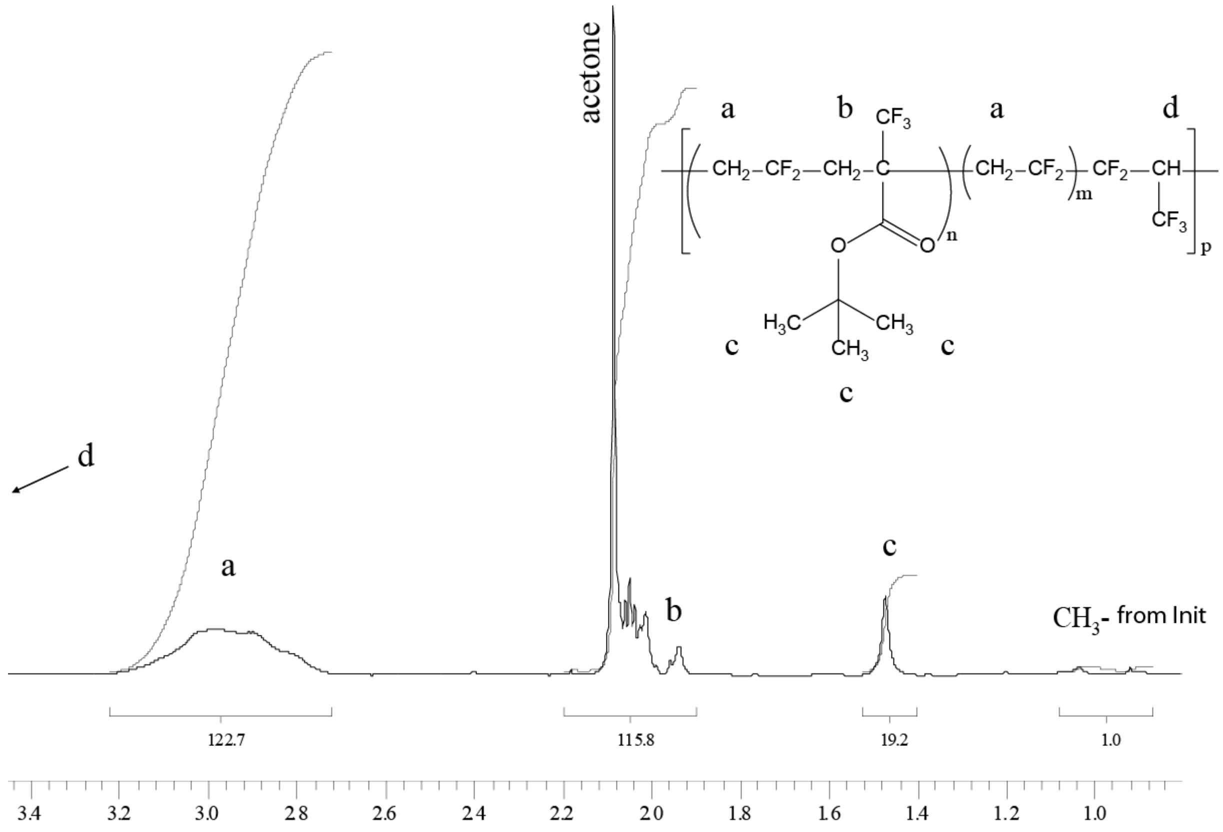

Figure S15: Expansion of the 0.8-3.4 ppm zone in the ^{1}H NMR spectrum of poly(PFP-*ter*-VDF-*ter*-MAF-TBE) terpolymers (recorded in acetone-d_6) (Runs 6-10 in Table 3)

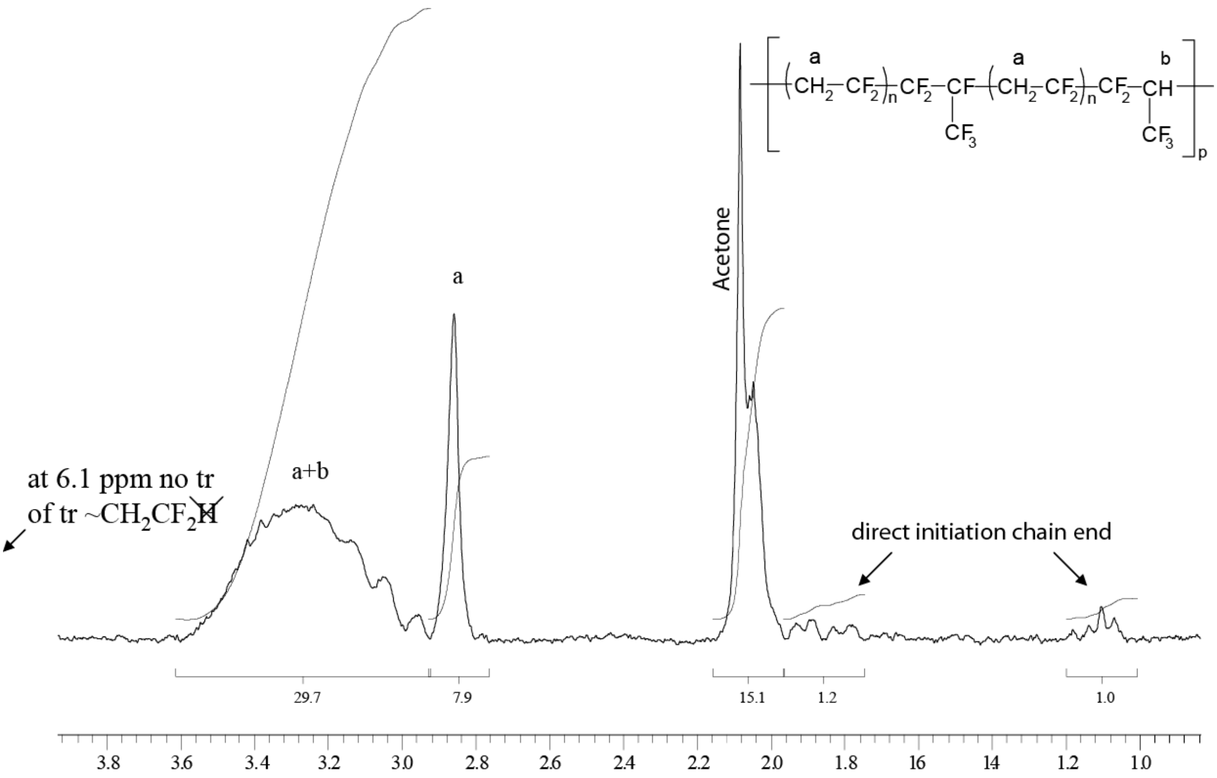

Figure S16: expansion of the 0.9-3.9 ppm zone of the ^{1}H NMR spectrum of poly(PFP-*ter*-VDF-*ter*-HFP) terpolymer recorded in acetone-d_6 (Run No 5, Table2)

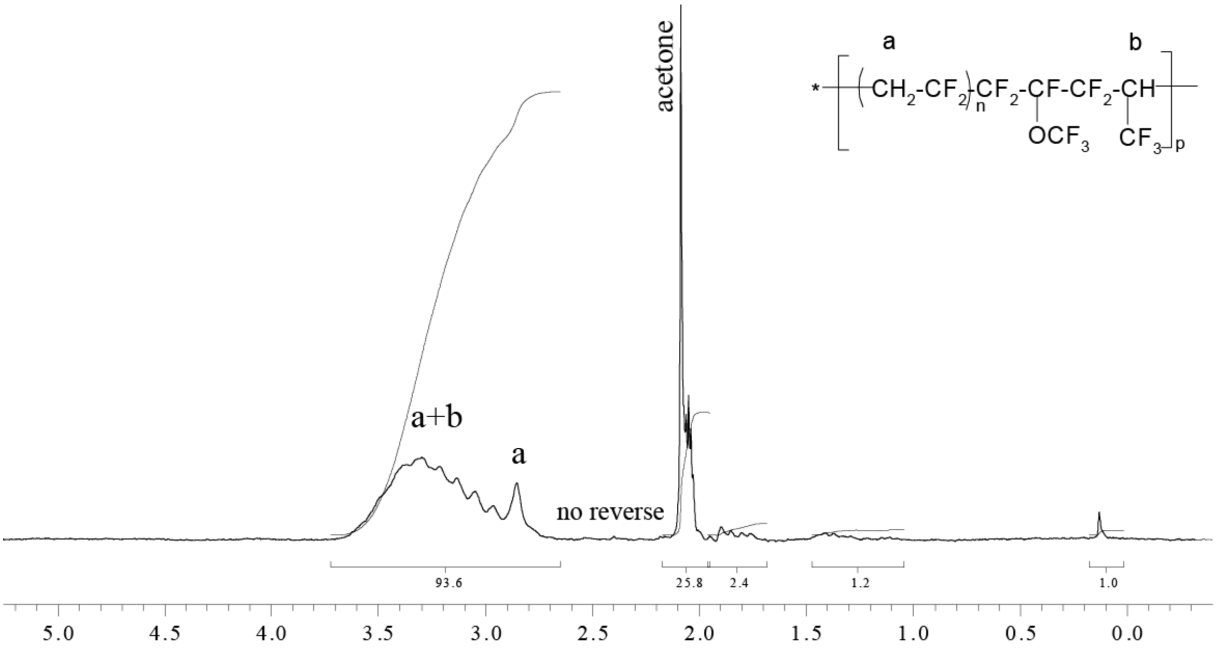

Figure S17: ^{1}H NMR spectrum of poly(PFP-*ter*-VDF-*ter*-PMVE) terpolymer (recorded in acetone d_6) (Run 8 in Table 2).

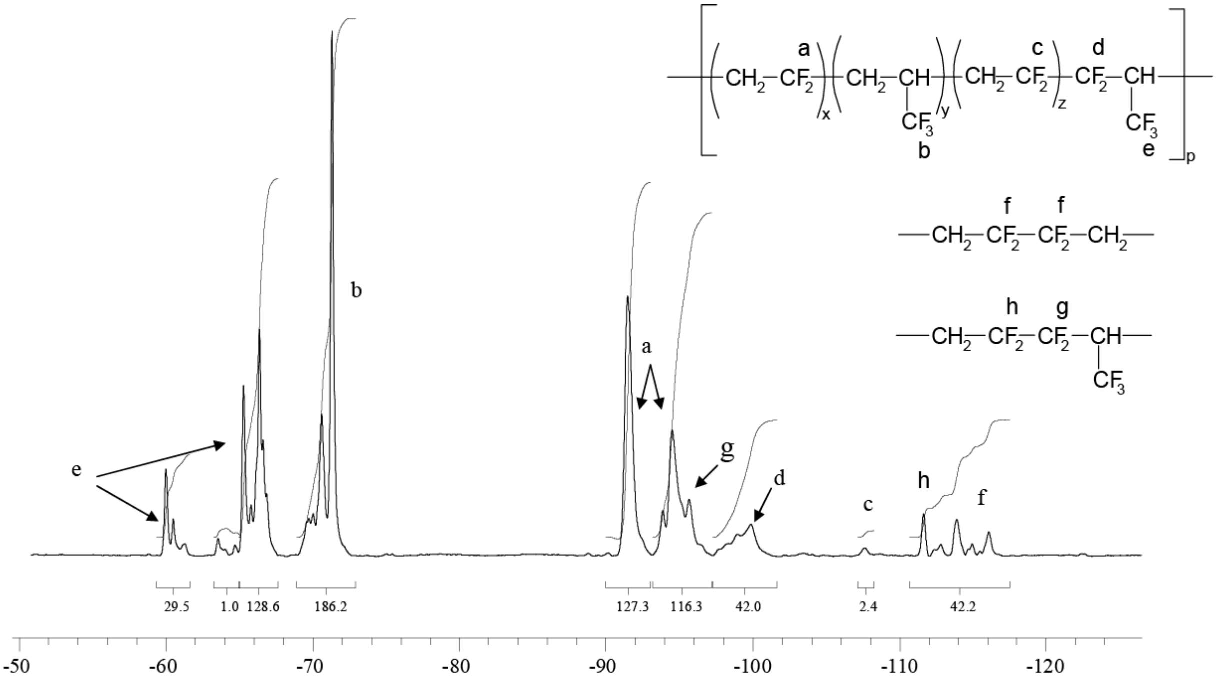

Figure S18: ^{19}F NMR spectrum of poly(PFP-*ter*-VDF-*ter*-TFP) terpolymer (recorded in acetone d6) (Run 10 in Table 2)

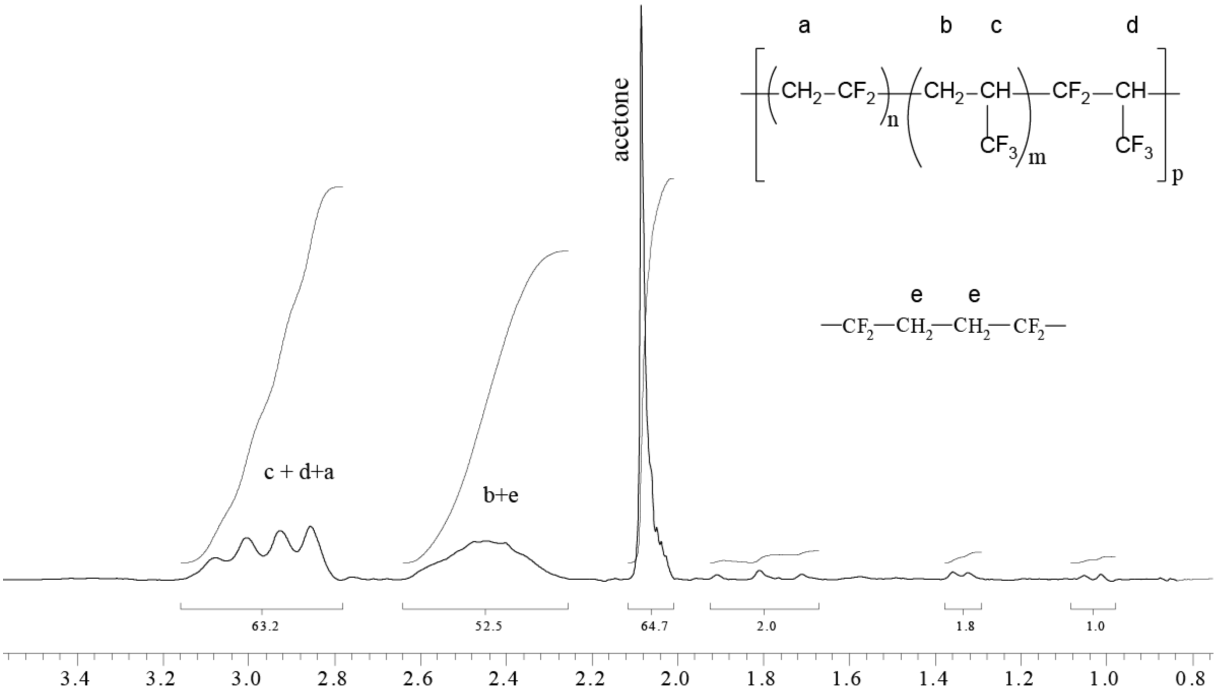

Figure S19: ^{1}H NMR spectrum of poly(PFP-*ter*-VDF-*ter*-TFP) terpolymer (recorded in acetone d6) (Run 10 in Table 2)

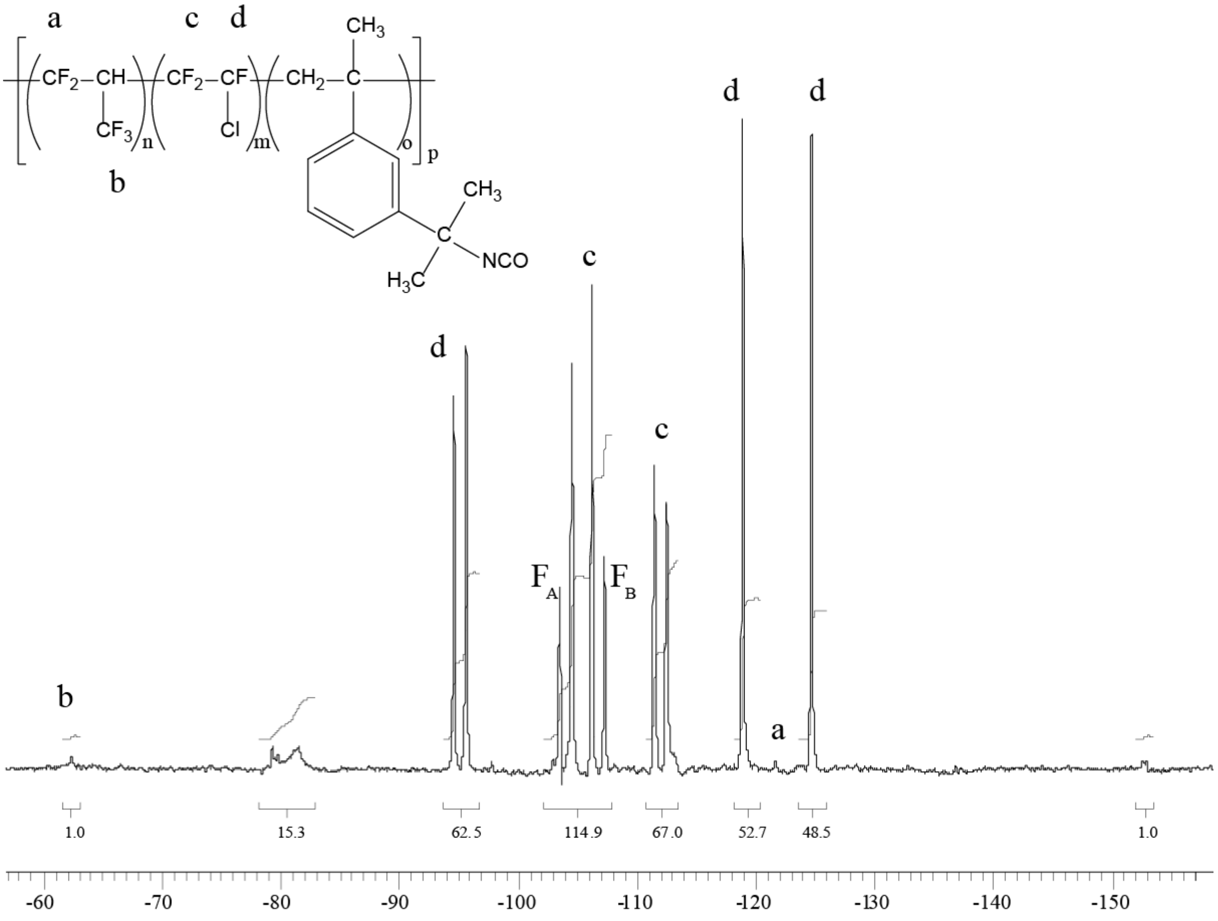

Figure S20: ^{19}F NMR spectrum of poly(PFP-*ter*-CTFE-*ter*-mTMI) terpolymer (recorded in acetone d6) (Run 3 in Table 4).

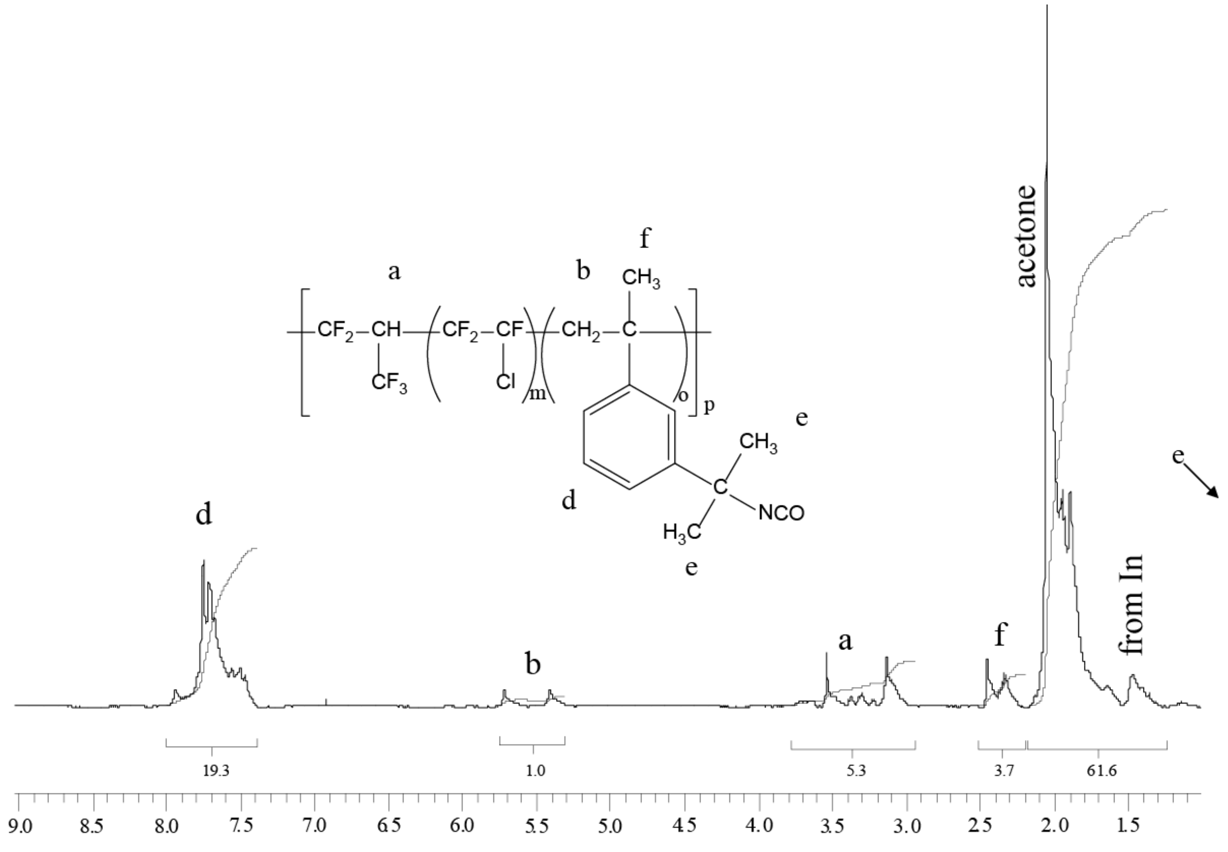

Figure S21: expansion of the 0.6 to 4.0 zone in the ^{1}H NMR spectrum of poly(PFP-*ter*-CTFE-*ter*-mTMI) terpolymer (recorded in acetone d_6) (Run 3 in Table 4)

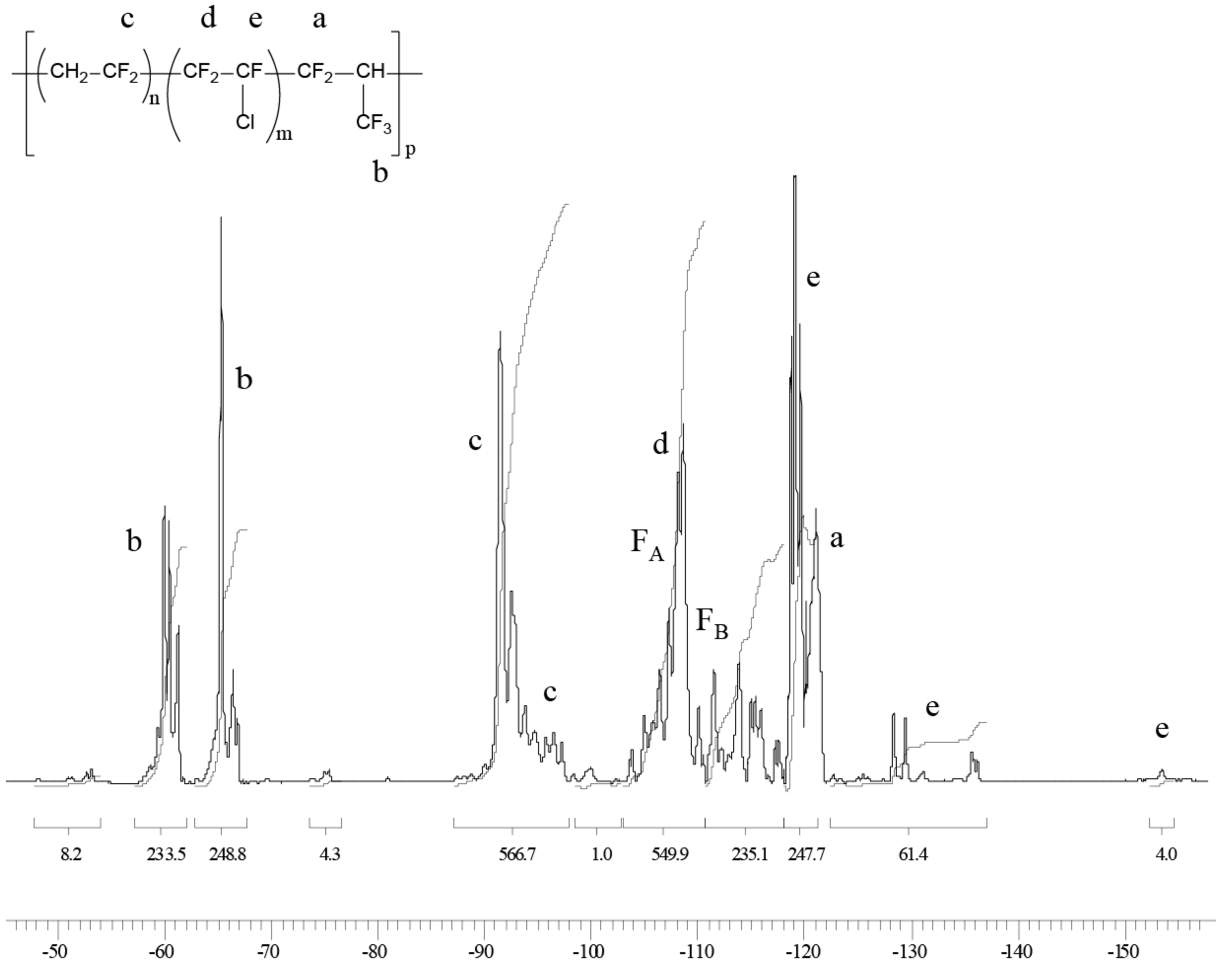

Figure S22: ^{19}F NMR spectrum of poly(PFP-*ter*-CTFE-*ter*-VDF) terpolymer (recorded in acetone d6) (Run 2 in Table 4)

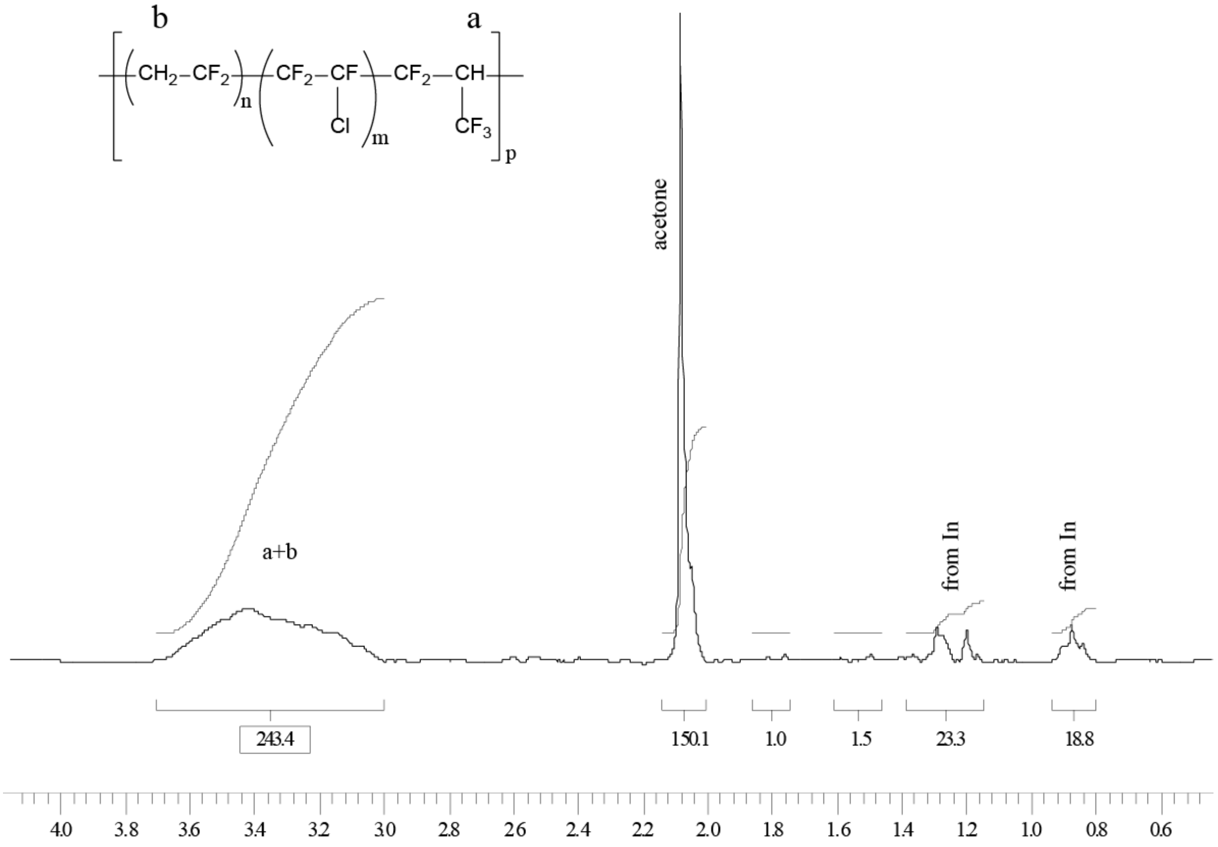

Figure S23: ^{1}H NMR spectrum of poly(PFP-*ter*-CTFE-*ter*-VDF) terpolymer, recorded in recorded in acetone d_6) (Run 2 in Table 4)

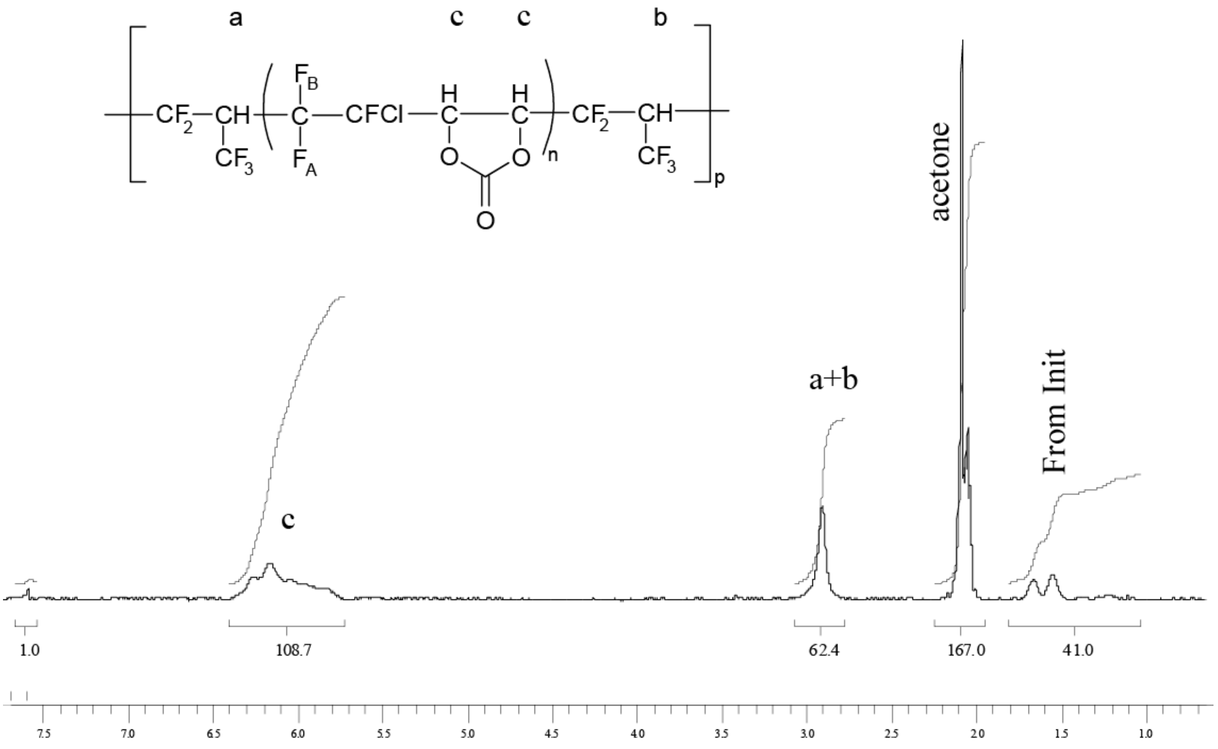

Figure S24: ^{1}H NMR spectrum of poly(PFP-*ter*-CTFE-*ter*-VCA) terpolymer (recorded in acetone d_6) (Run 4 in Table 4)

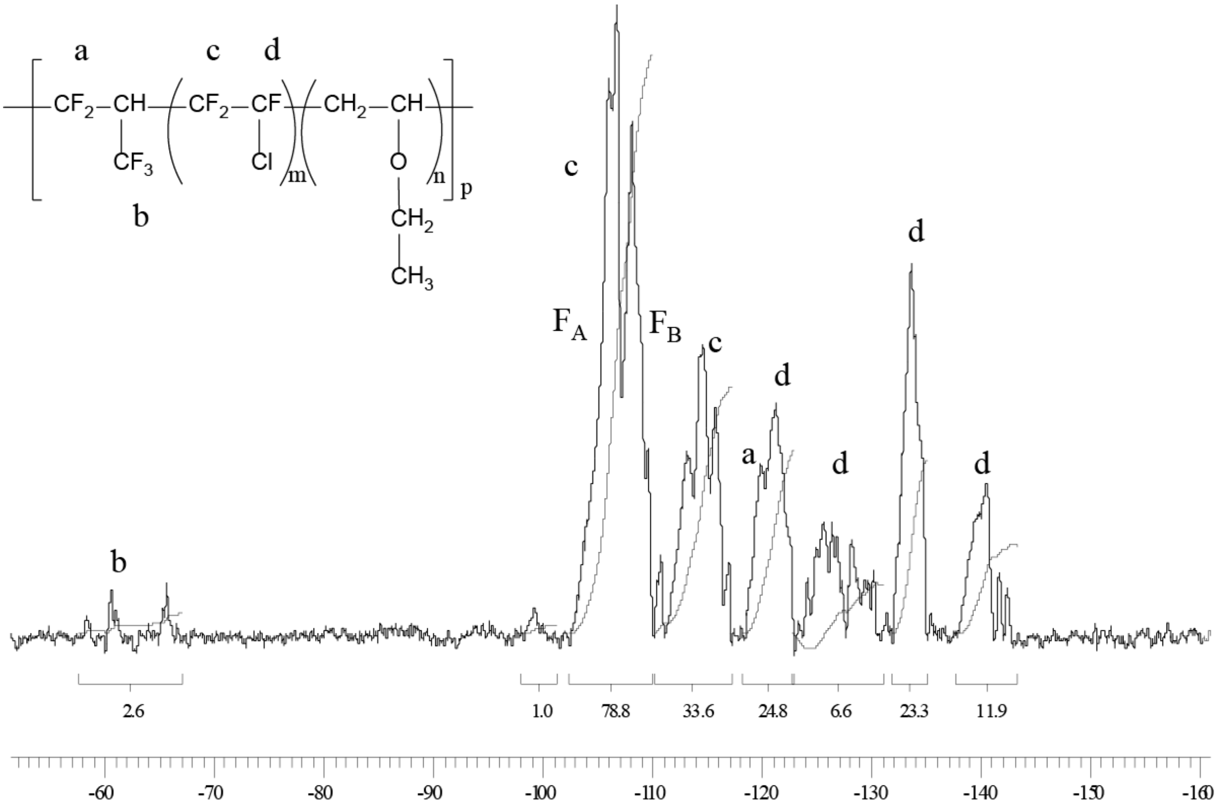

Figure S25: ^{19}F NMR spectrum of poly(PFP-*ter*-CTFE-*ter*-EVE) terpolymer (recorded in acetone d6) (Run 5 in Table 4)

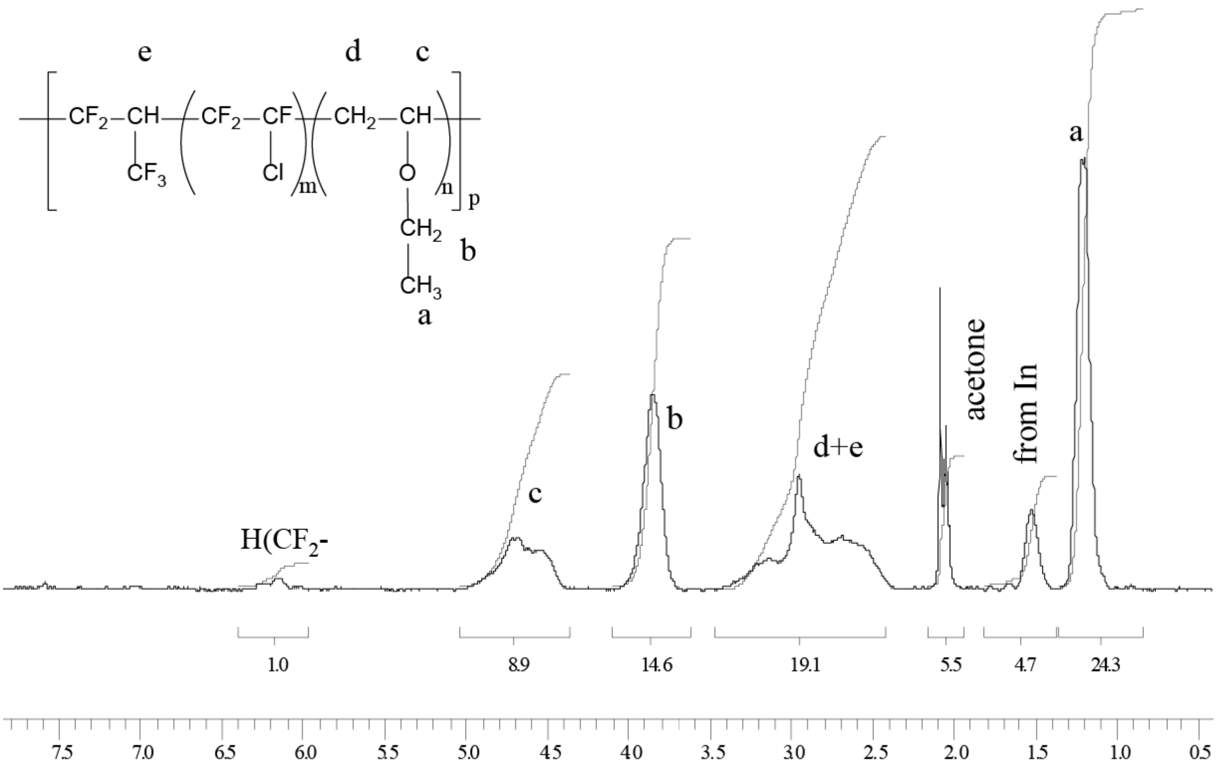

Figure S26: ^{1}H NMR spectrum of poly(PFP-*ter*-CTFE-*ter*-EVE) terpolymer (recorded in acetone d$_6$) (Run 5 in Table4)

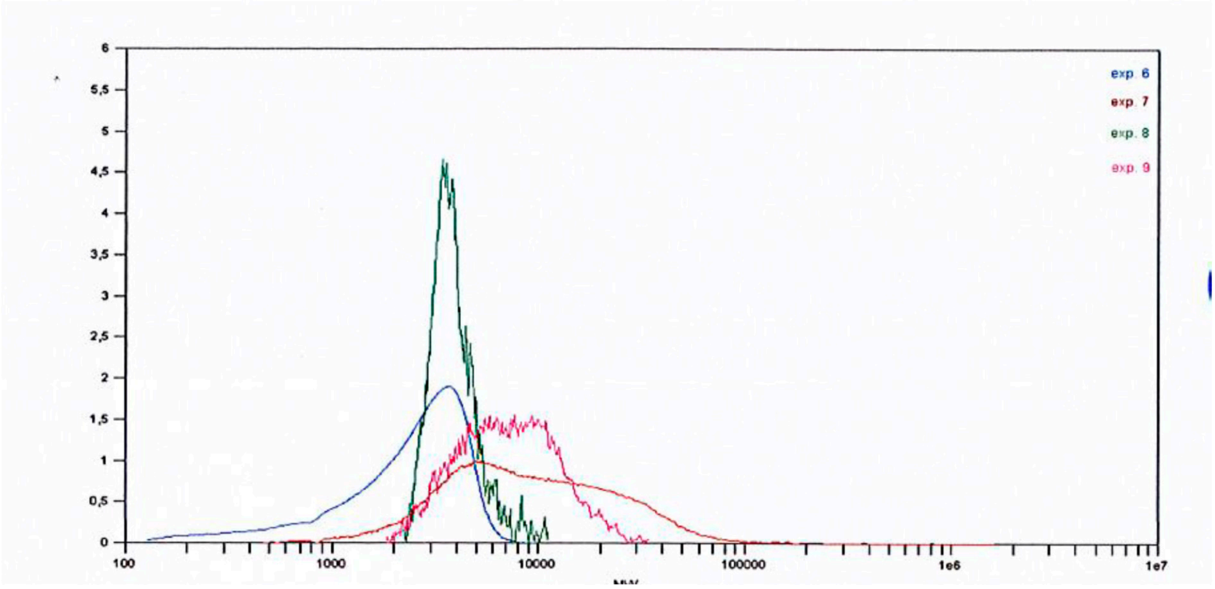

Figure S27: GPC chromatograms (Solvent THF, PMMA standards) of poly (PFP-*ter*-VDF-*ter*-MAF-TBE) terpolymers. The run numbers correspond to experiment numbers in Table 3.

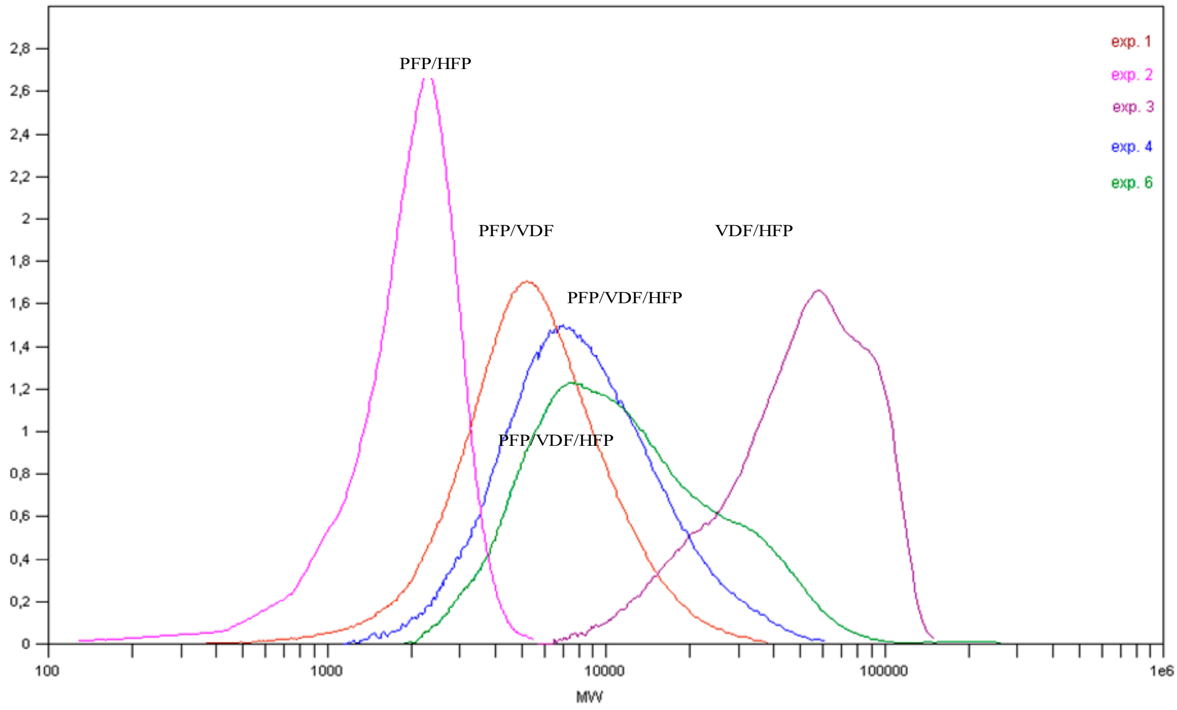

Figure S28: GPC chromatograms of co- and ter-polymers based on PFP, VDF and HFP

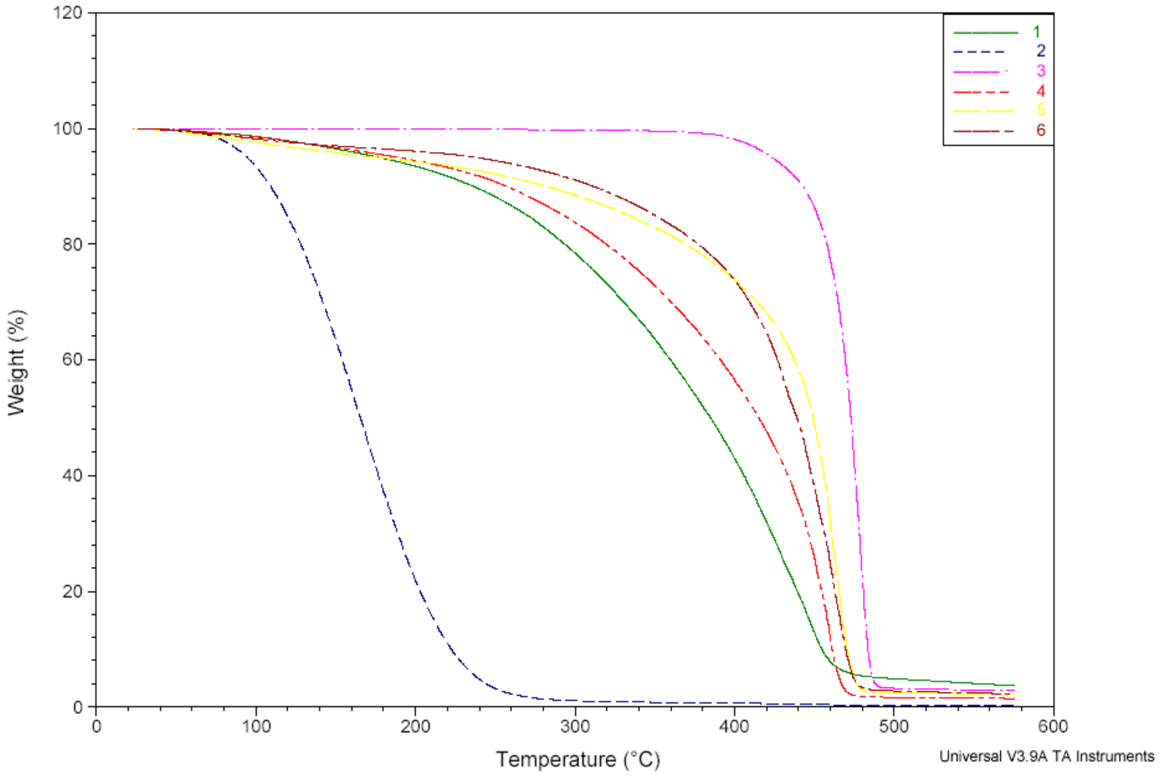

Figure S29: TGA thermograms under nitrogen of PFP/VDF, PFP/MAF-TBE, VDF/MAF-TBE copolymers. The number of the experiments of the caption corresponds to the number of experiments in Tables 1 and 3.

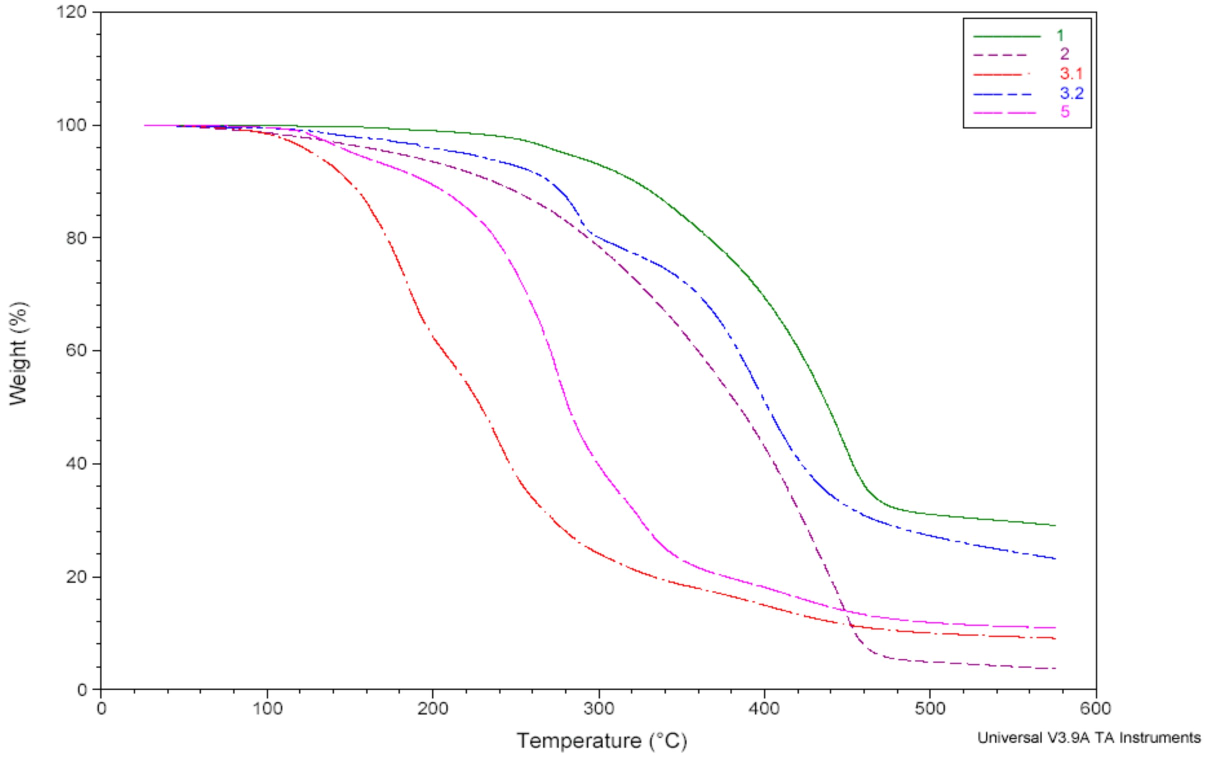

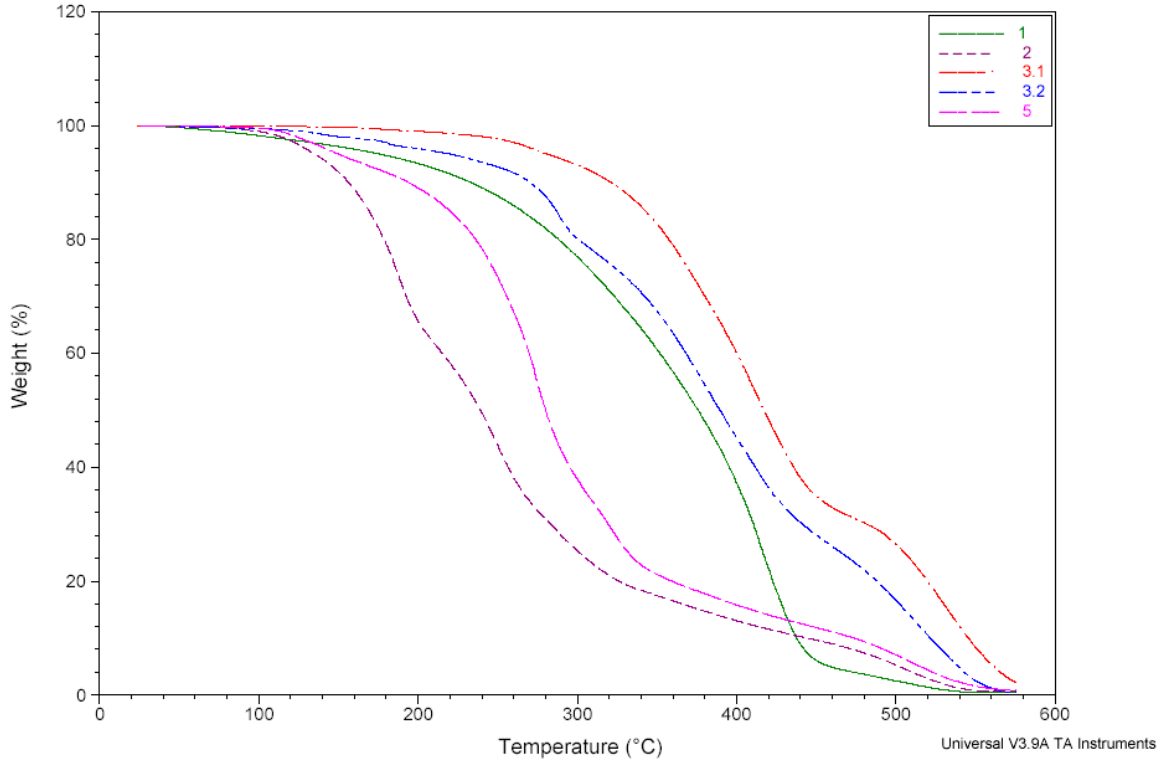

Figure S30: TGA thermograms under air of poly(PFP-*co*-VDF), poly(PFP-*co*-MAF-TBE), and poly(VDF-*co*-MAF-TBE) copolymers. The number of the experiments of the caption corresponds to the number of experiments in Tables 2-3.

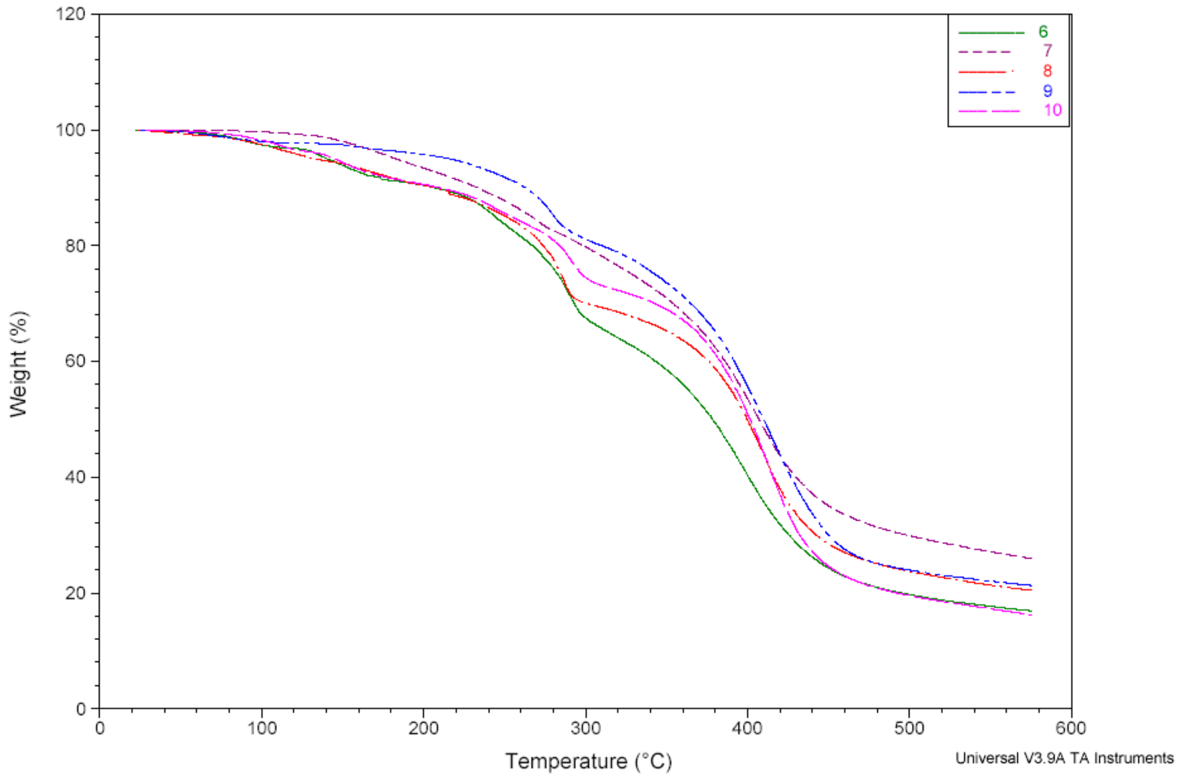

Figure S31: TGA thermograms under nitrogen of poly(PFP-*ter*-VDF-*ter*-MAF-TBE) terpolymers. The number of the experiments of the caption corresponds to the number of experiments in Tables 1 and 3.

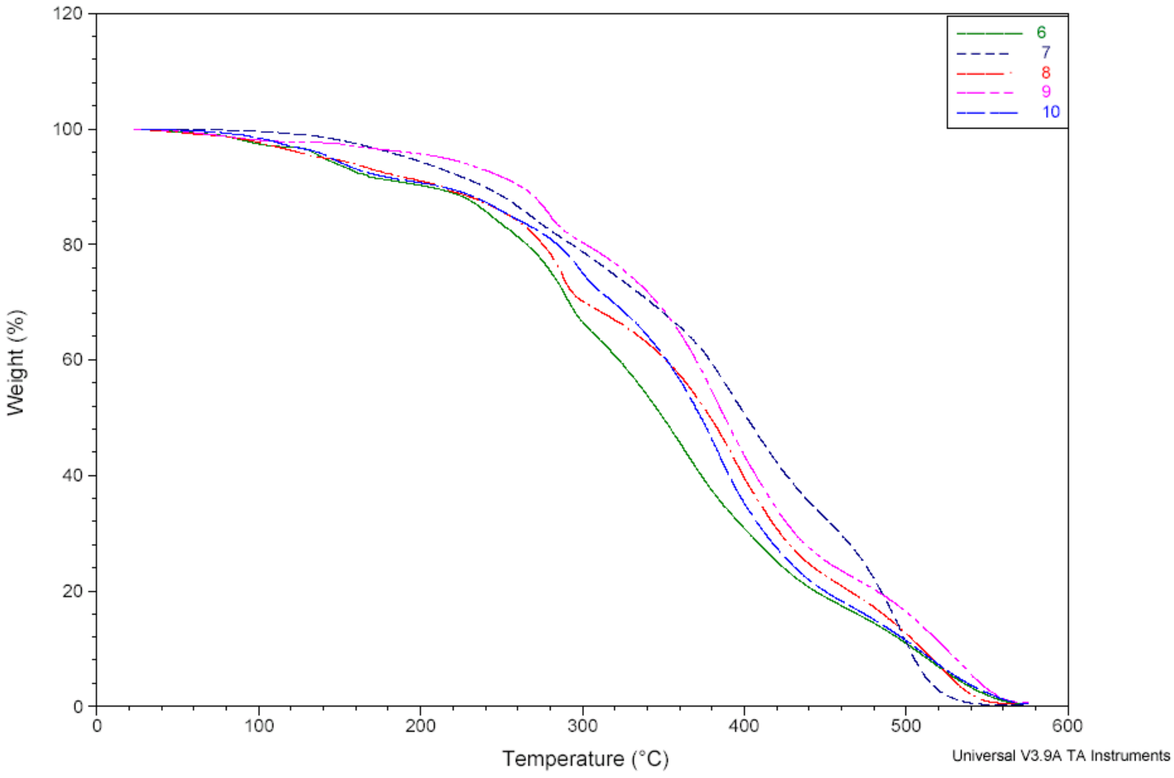

Figure S32: TGA thermograms under air of poly(PFP-*ter*-VDF-*ter*-MAF-TBE) terpolymers. The number of the experiments of the caption corresponds to the number of experiments in Tables 1 and 3.

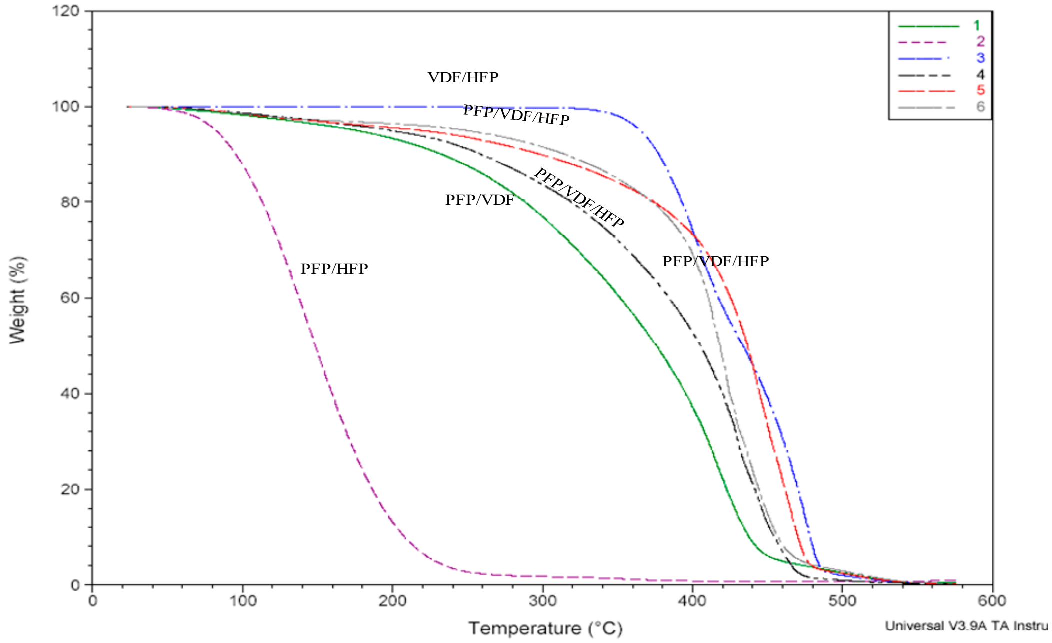

Figure S33: TGA thermograms under air of poly(PFP-*ter*-VDF-*ter*-HFP) co- and ter-polymers (Table 2).
